# Ultrafast Intersystem Crossing Dynamics of 6-Selenoguanine in Water

**DOI:** 10.1021/jacsau.2c00250

**Published:** 2022-06-28

**Authors:** Danillo Valverde, Sebastian Mai, Sylvio Canuto, Antonio Carlos Borin, Leticia González

**Affiliations:** † Department of Fundamental Chemistry, Institute of Chemistry, University of São Paulo, Avenida Professor Lineu Prestes, 748, São Paulo, São Paulo CEP 05508-000, Brazil; ‡ Institute of Physics, University of São Paulo, Rua do Matão 1371, São Paulo, São Paulo CEP 05508-090, Brazil; ⊥ Institute of Theoretical Chemistry, Faculty of Chemistry, University of Vienna, Währinger Straße 17, Vienna 1090, Austria

**Keywords:** nucleobase analogues, photobiology, intersystem crossing, nonadiabatic dynamics, ultrafast processes, QM/MM, transient absorption spectroscopy

## Abstract

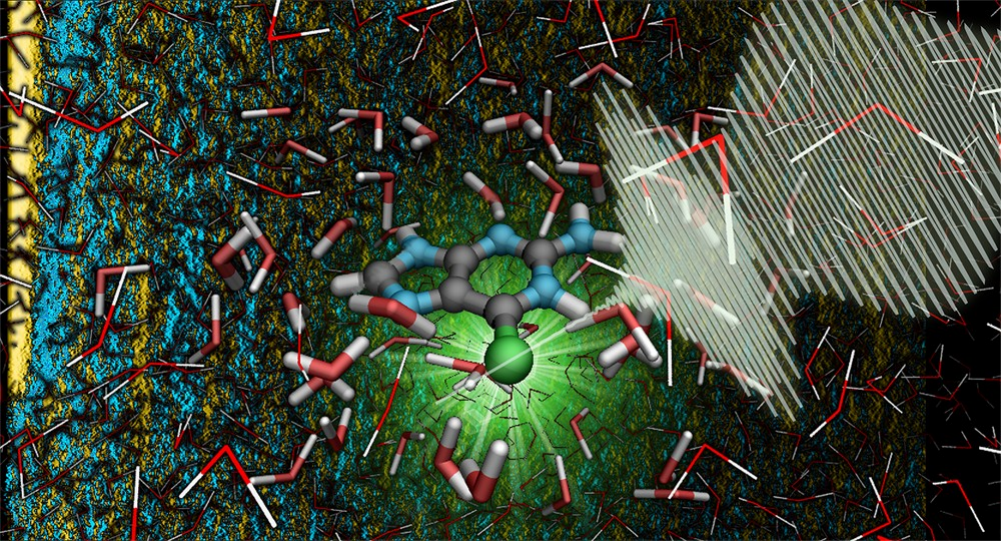

Rationalizing the photochemistry of nucleobases where an oxygen is replaced by a heavier atom is essential for applications that exploit near-unity triplet quantum yields. Herein, we report on the ultrafast excited-state deactivation mechanism of 6-selenoguanine (6SeGua) in water by combining nonadiabatic trajectory surface-hopping dynamics with an electrostatic embedding quantum mechanics/molecular mechanics (QM/MM) scheme. We find that the predominant relaxation mechanism after irradiation starts on the bright singlet S_2_ state that converts internally to the dark S_1_ state, from which the population is transferred to the triplet T_2_ state via intersystem crossing and finally to the lowest T_1_ state. This S_2_ → S_1_ → T_2_ → T_1_ deactivation pathway is similar to that observed for the lighter 6-thioguanine (6tGua) analogue, but counterintuitively, the T_1_ lifetime of the heavier 6SeGua is shorter than that of 6tGua. This fact is explained by the smaller activation barrier to reach the T_1_/S_0_ crossing point and the larger spin–orbit couplings of 6SeGua compared to 6tGua. From the dynamical simulations, we also calculate transient absorption spectra (TAS), which provide two time constants (τ_1_ = 131 fs and τ_2_ = 191 fs) that are in excellent agreement with the experimentally reported value (τ_exp_ = 130 ± 50 fs) (Farrel et al. *J. Am. Chem. Soc.***2018**, *140*, 11214). Intersystem crossing itself is calculated to occur with a time scale of 452 ± 38 fs, highlighting that the TAS is the result of a complex average of signals coming from different nonradiative processes and not intersystem crossing alone.

## Introduction

**Figure 1:**
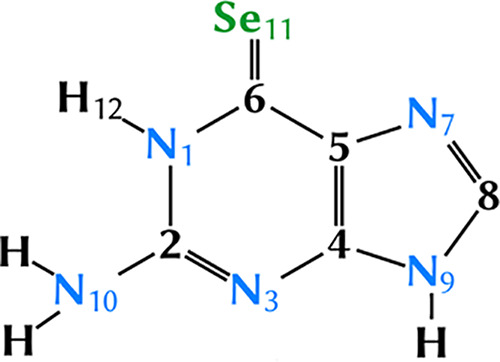
Schematic structure and atom numbering of 6-selenoguanine.

Natural evolution selected biomolecular building blocks resistant to photochemical damage. For instance, the nucleobases composing DNA and RNA show photochemical stability that is attributed to efficient radiationless deactivation to the electronic ground state.^1^ Internal conversion via accessible conical intersections (CIs) with the ground state safely dissipates the excess energy gained by excitation within a few picoseconds.^2−8^ Remarkably, the photochemical stability associated with canonical nucleobases is very sensitive to chemical modifications. For example, in chalcogen-substituted nucleobases, where an exocyclic carbonyl oxygen is replaced by heavier elements of the same group, internal conversion to the ground state is suppressed and singlet-to-triplet intersystem crossing (ISC) is enhanced up to near-unity quantum yields.^9−11^ The efficiency of ISC is such that some chalcogen-modified nucleobases are able to generate singlet oxygen (^1^O_2_) and thus find applications as photodynamic therapy photosensitization agents, antiviral and antimicrobial drugs, or in blood sterilization.^12,13^ Thio-analogues were the first to draw attention.^14−22^ For instance, 6-thioguanine (6tGua) is able to generate about 21% of ^1^O_2_.^14^ As selenium-substituted nucleobases are more stable in RNA^23^ and DNA^24^ than their sulfur counterparts and because they exhibit a red-shifted absorption spectrum,^25^ they could be even more appealing and effective drugs to treat deeper tissue carcinomas.^26^ However, their photochemistry is still puzzling. Computational chemistry is ideally suited to investigate photochemical processes in small organic compounds.^27^ And indeed, the exploration of potential energy surfaces (PESs) of nucleobase analogues with stationary quantum chemistry is a well-established research field,^6,7,28−31^ but nonadiabatic dynamical simulations are still much more scarce, particularly in solution.^32−36^ Herein, we set out to elucidate the relaxation dynamics of 6-selenoguanine (6SeGua, Figure 1) in aqueous solution, whose relaxation mechanism is still controversial.

On the basis of transient absorption spectroscopy (TAS), it was proposed that after UV photoexcitation, triplet states are populated much faster in 6SeGua than in the sulfur-counterpart (130 fs versus 350 fs, respectively)^25^ because of the heavy-atom effect. However, a shorter triplet-state lifetime was also measured in 6SeGua relative to 6tGua (1.7 ns versus 1420 ns, respectively). This means that less triplet population is available for reactions in 6SeGua than in 6tGua, counterintuitively making the former more photostable despite its higher ISC rate. A more recent TAS study on 6tGua with higher resolution reported that ISC process takes 522 fs,^37^ illustrating how difficult is to estimate correctly when the triplet states starts to be populated. Using 6tGua as a model, it was suggested^25^ that the S_2_^1^(ππ*) state deactivates to the S_1_^1^(nπ*) state, after which the triplet manifold could be populated via ISC to the T_2_^3^(nπ*) or to the T_1_^3^(ππ*) state. Using gas-phase multiconfigurational calculations, Cui and co-workers^38^ proposed two deactivation pathways to populate triplet states: a S_2_ → T_2_ and a S_1_ → T_1_ state—consistent with previous density functional theory (DFT) calculations of spin–orbit couplings (SOC).^39^ Recent QM/MM stationary calculations^40^ in water and in a DNA double strand indicate that after populating the bright S_2_ state, 6SeGua should decay to the S_1_ state much faster than in the gas phase because no barrier is present in solution. Then, from the S_1_ state, 6SeGua could undergo ISC to the triplet manifold because energetically accessible CIs with the electronic ground state were not found.^40^

With the aim to shed light on the origin of the experimental time constant previously attributed to ISC^25^ and to unequivocally find the predominant relaxation pathway of 6SeGua in solution, here we undertake a first-principles nonadiabatic dynamics study within a hybrid quantum mechanics/molecular mechanics (QM/MM) framework. The dynamics simulations serve to obtain the time constants of all the competing photochemical events that take place after irradiation but also to emulate electronic absorption and TAS signals of 6SeGua in water that can be directly compared with the experiment.^25^

## Methods

The QM/MM nonadiabatic dynamics simulations were carried out considering the 6SeGua in the QM part and the water molecules in the MM part. Previous static calculations on 6SeGua in the gas phase^38^ were obtained with multi-state complete-active-space second-order perturbation theory (MS-CASPT2);^41−44^ however, no dynamic simulations at this level of theory are possible. Therefore, we first investigated the suitability of the more cost-efficient algebraic diagrammatic construction scheme for the polarization propagator to the second-order (ADC(2))^45−49^ method, by comparing the PESs of 6SeGua for the relevant singlet and triplet excited states at both levels of theory in the gas phase.

### Gas-Phase Stationary Electronic Structure Calculations

MS-CASPT2 and ADC(2) excited-state calculations were done with the cc-pVDZ^50^ basis set on a ground-state equilibrium geometry optimized at the MP2/cc-pVDZ level. Ground- and excited-state calculations (Tables S1–S3) including scalar relativistic effects via second-order Douglas–Kroll–Hess formalism (DHK2)^51^ and the ANO-RCC-VDZP basis set^52^ agree well with results computed with the cc-pVDZ basis set excluding relativistic corrections. SOC and vertical excitations energies calculations with the cc-pVDZ, and cc-pVDZ-DK were also performed, and they likewise showed a good agreement between the employed atomic basis sets. These results and previous work^53^ justify the use of the cc-pVDZ basis set.

**Figure 2:**
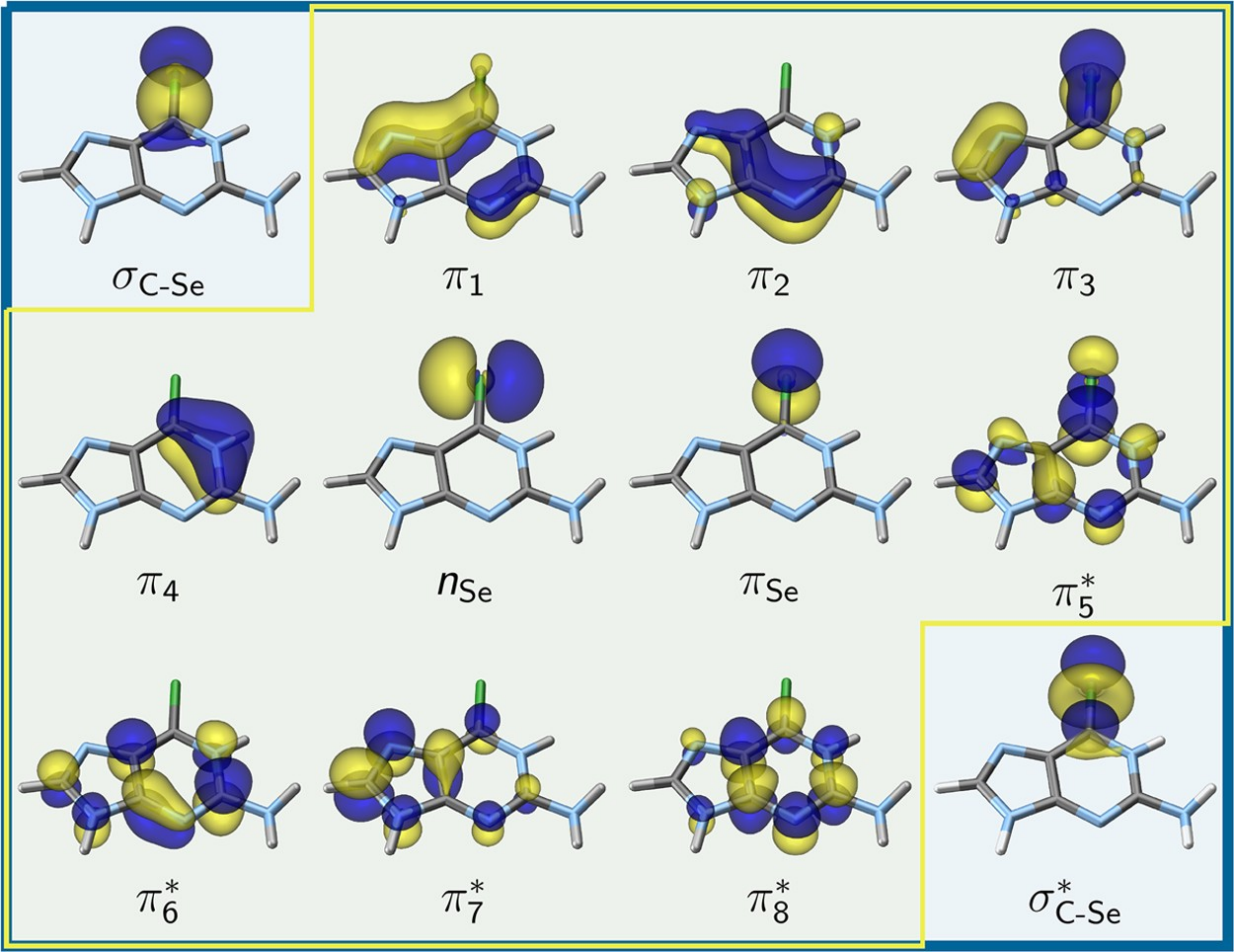
Active spaces used during the MS-CASPT2/cc-pVDZ calculations. Yellow boxed orbitals were used for geometry optimizations (CAS(12,10)) and blue boxed ones for vertical singlet point energies (CAS(14,12)).

The orbitals included in the active space of the MS-CASPT2 were selected from state-average complete active space self-consistent-field (CASSCF) test calculations over the four lowest-lying singlet states. Such calculations allowed us to exclude two π orbitals and the two nitrogen lone-pairs (*n*_N_) with occupation numbers very close to two. Thus, these orbitals were kept in the inactive space at the CASSCF level, but correlated at the MS-CASPT2 level for recovering most of the dynamical correlation effects. The final active space is then composed of nine π and π* orbitals plus the *n*_Se_ lone-pair orbital located on the selenium atom (denoted as CAS(12,10) in Figure 2), as in previous work.^38^ Additional tests at the ADC(2)/cc-pVDZ level of theory (Table S4) showed that the first electronic transition involving the *n*_N_ orbitals lies about 2 eV above the experimental excitation window, additionally supporting the exclusion of such orbitals.

Singlet and triplet minima were optimized using numerical gradients at the MS-CASPT2/cc-pVDZ level. For the optimization of the critical points, four singlet and three triplet states were averaged with equal weights. However, as *OpenMolcas*(54) does not average over singlet and triplet states simultaneously, the states of each multiplicity were calculated separately. At each optimized structure, final energies were recomputed by augmenting the previous active space with the σ and σ* orbitals located on the C–Se moiety, resulting in the CAS(14,12) active space (Figure 2). To deal with intruder states, we used an imaginary level shift^55^ of 0.2 a.u. and employed no IPEA^56,57^ shift correction. The RICD technique^58^ was employed to speed up the two-electron integral calculation. All the MS-CASPT2 calculations were carried out with the *OpenMolcas 18* suite.^54^

The singlet and triplet minima energy points were then reoptimized at the ADC(2)/cc-pVDZ level with analytical gradients and the RI approach, as implemented in *Turbomole 7.0*.^59^ The ground state was computed with MP2, whereas the excited states used ADC(2) based on the MP2 ground-state wave function. Several minimum energy crossing points related to CIs and singlet–triplet crossing points were optimized with both the MS-CASPT2 and ADC(2) methods. As neither method provides nonadiabatic coupling vectors, we used the penalty function procedure proposed by Levine, Coe, and Martínez^60^ for minimum energy crossing points optimization. In the case of singlet–triplet crossings, such vectors are not needed; thus, we adopted the Bearpark–Robb–Schlegel algorithm.^61^ The optimizations were performed using the interface between the *SHARC*(62) (*Surface Hopping including ARbitrary Couplings*) code and the external *ORCA 4.0.1*(63) optimizer.

The obtained structures were connected with linear interpolation in internal coordinates (LIIC) paths, which allow us to check for consistent active space composition and for the existence of energetic barriers along the pathway. We note that any barrier in a LIIC pathway is an upper bound to the true reaction barrier. Therefore, if a LIIC scan does not show a barrier, then no barrier exists between the two end points.

Depending on the level of theory, the SOC elements were computed with different formalisms. The atomic mean-field integrals^64^ and the spin–orbit restricted active space state interaction approach^65^ is employed for the MS-CASPT2 calculations, using the perturbatively modified CASSCF wave functions. In the ADC(2) calculations, the SOC elements were computed in *Turbomole*(66) using spin–orbit mean field integrals from *Orca 4.0.1*.^63^ The effective SOCs reported herein are given as follows:
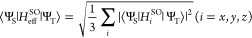
1where Ψ_S_ and Ψ_T_ are, respectively, the electronic wave functions of the corresponding singlet and triplet states, and *H*_*i*_^SO^ (*i* = *x, y, z*) are the components of the spin–orbit operator.

All the comparisons of ADC(2) against MS-CASPT2 results are done in the gas phase, under the assumption that if this agreement is reasonable, ADC(2) should also reproduce MS-CASPT2 reasonably well in solution when this is taken care explicitly with QM/MM, as described below. For reference, we mention here that according to the optimization calculations, ADC(2) using analytical gradients is about 40–50 times faster than MS-CASPT2 using numerical gradients.

### QM/MM Setup and Initial Conditions

Solvated 6SeGua was first modeled through classical molecular dynamics (MD) simulations from the *AMBER*(67,68) software with periodic boundary conditions (PBC). Solute atomic charges (Table S5) were obtained by fitting the electrostatic potential according to the RESP (restrained electrostatic potential)^69^ procedure at the B3LYP/cc-pVDZ level of theory, as implemented in the *Gaussian 09* package.^70^ In this calculation, the MP2/cc-pVDZ optimized ground-state minima of the isolated 6SeGua was used as a reference. Intramolecular and intermolecular interactions of the solute (except the parameters associated with the selenium atom) were modeled employing the generalized *AMBER* force field (GAFF).^71^ Selenium atom parameters (reproduced in Listing S1) were obtained from a parametrization of 2-selenouridine embedded in aqueous solution.^72^

**Figure 3:**
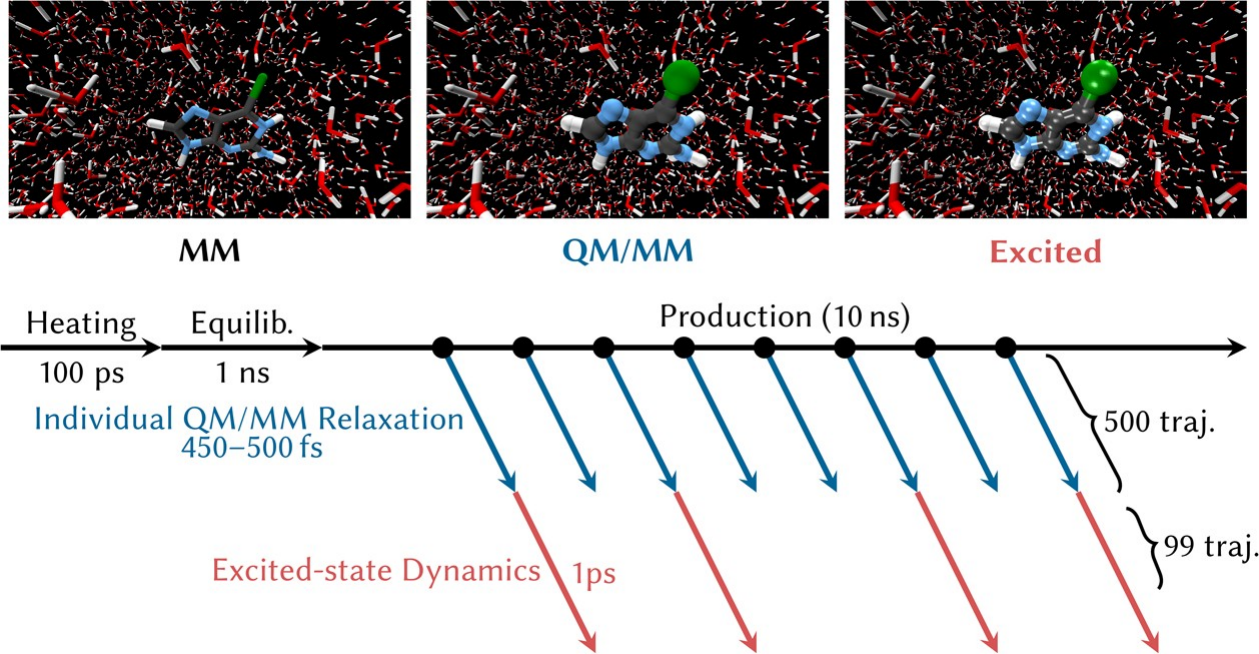
Generation of initial conditions to be used in nonadiabatic QM/MM dynamics simulations.^74^ First, classical molecular mechanics simulations, including heating, equilibration, and production were performed, from which 500 snapshots were selected. Subsequently, 500 QM/MM ground dynamics simulations were carried out to re-equilibrate the geometries on QM/MM level (blue). Finally, after stochastic excitation, nonadiabatic dynamics was performed for 99 trajectories (red).

In the classical MD calculations we employed a large simulation box to ensure proper solvation of the solute, as the subsequent nonadiabatic dynamics simulations in *SHARC*(62) currently cannot be done with PBC. Accordingly, 6SeGua was placed in a 30 Å truncated octahedron simulation box containing 5406 water molecules represented by the flexible three-point fixed-charge water model SPC/Fw.^73^ We chose a flexible water model with no constraints because we use a time step of 0.5 fs in all the MD simulations (as needed later for the nonadiabatic dynamics simulations). At the beginning (Figure 3), the total energy of the system was minimized employing the steepest descent algorithm to remove bad contacts and cavities in the generated solvent box. After that, a 100 ps heating step was performed in the NVT ensemble, scaling the temperature from 0 to 300 K. An equilibration of the system was performed over 1 ns in the NPT ensemble in standard conditions (300 K and 1 bar) using the Langevin thermostat. Finally, a 10 ns production run was done, from which 500 configuration snapshots were selected with 20 ps spacing and turned into *SHARC* format as detailed elsewhere.^74^ The last step includes the back-propagation of the coordinates by half a time step, as *AMBER* and *SHARC* store coordinates and velocities differently.^74^

On the basis of previous work,^74,75^ for each of the 500 snapshots, a short QM/MM-MD trajectory in the ground state was carried out to allow the geometry to relax from the approximate force-field distribution to the ab initio distribution. These and subsequent steps were performed without PBCs, after reimaging all atoms to the primary box. The simulation time of this QM/MM dynamics (450–500 fs) was randomly determined to avoid bias at the instant of the excitation, as otherwise all trajectories would evolve coherently to the same geometries in the way to adapt from the classical force field to QM/MM forces.^74^ Although the solvent distribution around the solute cannot be fully relaxed during this short time, we assume that the RESP charges used in the MM MD represent the electron density reasonably well. The QM/MM simulations were performed with the *SHARC 2.1* program.^62,76^ The computation of the MM forces is carried out with the *Tinker* program^77^ and the QM region is calculated at the MP2/cc-pVDZ level with the *Turbomole* program.^59^ The interaction between the QM and MM regions is described by an electrostatic embedding scheme, where the MM point charges directly interact with the electron density. This technique has been shown to work well in nucleobases.^78−80^

### Nonadiabatic QM/MM Dynamics Simulations

The end points of each of the 500 QM/MM ground-state trajectories were used as initial conditions in the nonadiabatic dynamics as follows. For each of the 500 snapshots, we calculated vertical excitation energies and oscillator strengths (from the electric transition dipole moment in length representation) using 15 singlet states at the ADC(2)/MM level of theory to produce an absorption spectrum covering the first two absorption bands. Additionally, 14 triplet states were computed at the same level to inspect which of these states need to be considered in the dynamics. The electronic absorption spectrum for 6SeGua in water is then obtained as a sum over Gaussians with full width at half-maximum of 0.25 eV centered at the computed vertical excitation energies with heights proportional to the oscillator strength. We note that the inclusion of SOCs between the 15 singlet states and the 14 triplet states does not visibly affect the shape of the absorption spectrum (Figure S1). The initial excited states were selected stochastically using the oscillator strength^81^ in the 3.45–3.59 eV (345–359 nm) window, covering the experimental excitation window employed by Farrell et al.^25^ This energy window contained 90 S_0_ → S_1_ and 152 S_0_ → S_2_ transitions. Among these 242 transitions, the highest oscillator strength was given a selection probability of 100%, and the other transitions were assigned probabilities proportional to the oscillator strength. The stochastic selection yielded 136 initial conditions (from which 20 start in the S_1_ and 116 in the S_2_). From them, we propagated 100 trajectories (15 from the S_1_ and 85 from the S_2_) for 1 ps. One trajectory was discarded due to numerical instabilities, so all analyses presented below were based on 99 trajectories.

The nonadiabatic dynamics simulations were performed with the *SHARC* method,^76^ an extension of Tully’s^82^ trajectory surface hopping able to describe internal conversion and ISC on the same footing. We included the S_0_, S_1_, and S_2_, as well as T_1_, T_2_, and T_3_ states, based on the chosen excitation energy range and an inspection of the density of triplet states within the 500 vertical excitation calculations. Note that in *SHARC*, the individual *M*_S_ components of the triplet states are explicitly included, meaning that the simulations considered a total of 12 electronic states.

The nuclear time step was 0.5 fs. The electronic wave function was evolved in time using the local diabatization procedure^83^ with a time step of 0.02 fs.^76^ Nonadiabatic couplings were obtained via function overlaps computed with the *WFoverlap* program,^84^ truncating the one-electron part of the excited-state wave functions^49^ to 99.95% of their norm.^84^ To correct for overcoherence, we applied an energy-based electronic decoherence correction^85^ with a parameter of 0.1 au to the populations of the spin-mixed electronic states, considering only the kinetic energy of the solute. After a hop, the velocities of the solute were rescaled to conserve total energy.

Trajectories were analyzed in terms of the electronic spin-free populations,^76^ charge transfer character (using *TheoDORE*(86)), and relevant geometric parameters. We also simulated the TAS, not at every time step, but at the times −0.20, −0.08, 0.00, 0.08, 0.20, and 0.60 ps. To this end, at these times, we performed additional ADC(2) single-point calculations including a total of 15 singlets and 13 triplet states that allow us to cover the experimental probe range of 400–700 nm (1.4–3.0 eV)^25^ and additionally the range of 300–400 nm (3–4 eV) that was not measured previously.^25^ The electronic Hamiltonian including the energies of these states and the SOCs of the S_0_–S_2_ and T_1_–T_3_ states was then constructed and diagonalized to obtain a set of states consistent with the electronic states in the trajectory (such that the active state of the trajectory is contained in this set) and additional high-energy states. The TAS was evaluated from the energy differences and oscillator strengths (from the electric excited-state-to-excited-state transition dipole moment^87^ in length representation) between active state and all other states, where we included both singlet–singlet and triplet–triplet transition dipole moments. The final TAS was generated from all trajectories by convolution with two-dimensional Gaussian functions with full width at half-maximum of 0.25 eV (to produce a realistic bandwidth from the limited amount of data) times 200 fs (to reproduce the instrument response function of the experiment^25^). To also include the effect of ground-state bleach into the TAS, we subtracted the properly scaled ground-state absorption spectrum, multiplied with a temporal error function, from the simulated TAS.

The solvent organization around the solute was determined by means of the pairwise radial distribution functions (*g*(*r*)).^88^ The ground state *g*(*r*) was evaluated from all 500 MD snapshots, using a bin size of 0.3 Å. The *g*(*r*) in the excited state was averaged over all trajectories. The resulting *g*(*r*) evidence no issues with droplet evaporation at distances below 25 Å from the solute.

## Results and Discussion

### Excited States in the Gas Phase

A detailed comparison of the vertical excitation energies, critical points, and deactivation pathways in the gas phase with ADC(2) versus previous MS-CASPT2 results^38^ can be found in Section S3. Here, only a short summary of the excitation energies is given to facilitate the upcoming discussion.

Table 1 displays the gas-phase vertical excitation energies of 6SeGua, computed at different levels of theory. All methods predict the lowest-lying singlet excited state to be an excitation from the lone pair localized on the Se atom (*n*_Se_) to the π_5_^*^ antibonding orbital (^1^(*n*_Se_π_5_^*^))—similar to the 2-selenouracil (2SeUra) ^1^(*n*_Se_π_2_^*^) state.^53^ At MS-CASPT2(14,12), the bright S_2_ state, ^1^(π_Se_π_5_^*^), is located at 3.45 eV, in good agreement with the experimental value.^25^ The ADC(2) (3.61 eV) and TD-B3LYP (3.60 eV) values are closer to MS-CASPT2(12,10), but the deviation from the experiment is acceptable. Similarly, the energy of ^1^(π_Se_π_6_^*^) obtained with ADC(2) is close to that from MS-CASPT2(12,10). The two lowest triplet states are roughly located at 2.5 and 2.7 eV, regardless of ADC(2) or MS-CASPT2 levels, with small deviations. Relevantly, all low-lying electronic states involve electronic excitations from selenium orbitals, whereas excitations localized on the nitrogen atoms or on the ring appear at much higher energy (>4.9 eV, see Table S5) and so do not take part in the photophysical events considered here. In general, deviations between ADC(2) and MS-CASPT2 are within 0.1–0.2 eV.

**Table 1:** 6SeGua Gas-Phase Vertical Excitation Energies (eV) (Oscillator Strength in Parentheses) Computed at the MS(4,3)-CASPT2(12,10)/cc-pVDZ, MS(4,3)-CASPT2(14,12)/cc-pVDZ, and ADC(2)/cc-pVDZ Levels of Theory; Experimental Data and Previous TD-B3LYP Calculations Are Reported for Comparison

state	TD-B3LYP^39^	MS-CASPT2(12,10)	MS-CASPT2(14,12)	ADC(2)	exp^25^
^1^(*n*_Se_π_5_^*^)	3.1	2.83 (0.000)	2.76 (0.000)	2.76 (0.000)	
^1^(π_Se_π_5_^*^)	3.6 (0.460)	3.73 (0.360)	3.45 (0.472)	3.61 (0.404)	3.47
^1^(π_Se_π_6_^*^)		4.64 (0.047)	4.34 (0.054)	4.64 (0.042)	
^3^(π_Se_π_5_^*^)	2.5	2.46	2.43	2.55	
^3^(*n*_Se_π_5_^*^)	2.9	2.74	2.67	2.63	

ADC(2) also faithfully reproduces the SOC matrix elements from MS-CASPT2 computed for the nearly planar ground-state geometry (Table S3). Obeying the El-Sayed rule,^89^ a large SOC value is obtained between states of different molecular orbital types (S_1_ → T_1_ and S_2_ → T_2_), whereas smaller SOCs are obtained if both states have the same orbital type (S_1_ → T_2_ and S_2_ → T_1_). As shown in Table S3, ADC(2) does seem to predict slightly larger “small SOCs”, but this is due to slightly different nπ*–ππ* mixing between ADC(2) and MS-CASPT2 at the used geometry. We expect that across all trajectories, both methods produce qualitatively similar SOCs and therefore the ISC time constants are reasonably predicted by ADC(2).

Representative geometries of gas-phase-optimized critical points and minimum energy crossing points are shown in Figure S2 and relevant conformational parameters and relative energies are collected in Table S6. Further, Figure S3 depicts the evolution of the S_0_, S_1_, S_2_, T_1_, and T_2_ states along the LIIC photochemical pathways. The satisfactory agreement of ADC(2) against our best level of theory (MS-CASPT2(14,10)) in the vertical excitation energies, critical point structures, and excited-state deactivation pathways justifies its use in the nonadiabatic dynamics simulations. We note that ADC(2) is formally a single-reference method that cannot correctly describe S_0_/S_*n*_ conical intersections,^90^ but correctly describes conical intersections between two excited states.^47^ In 6SeGua, the relaxation dynamics does not involve the S_0_/S_*n*_ conical intersection (see Figure S3), and thus all relevant intersection topologies are adequately represented by ADC(2) and no multireference method is required.

### Electronic Absorption Spectrum Simulation

**Figure 4:**
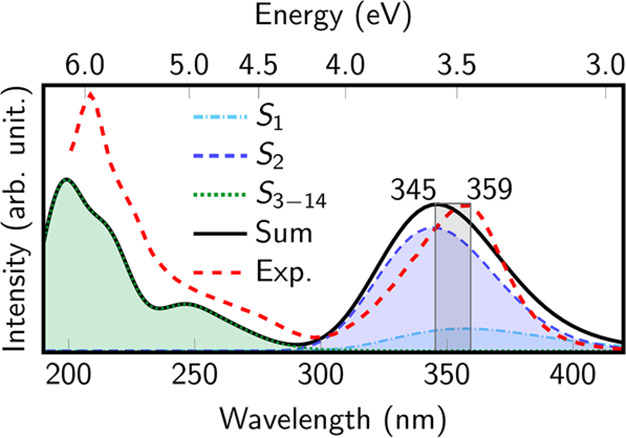
Electronic absorption spectrum of 6SeGua in water based on 500 snapshots from the QM/MM simulations in the ground state, computed at the ADC(2)/cc-pVDZ level. The gray region denotes the excitation window (3.45–3.59 eV; 345–359 nm) considered in the initial conditions selection process for the nonadiabatic dynamics calculations. See Figure S4 for a simulated spectrum including SOC.

The 500 snapshots obtained from the QM/MM MD ground-state dynamics were utilized to produce an absorption spectrum of 6SeGua in water, see Figure 4. This spectrum exhibits an absorption band ranging from 3–4 eV and peaking at 3.59 eV (345 nm), with contributions from the S_1_ and S_2_ singlet excited states, where the S_2_ contributes predominantly. This indicates that at most geometries the S_2_ is predominantly of ππ* character, although in water the gap between nπ* and ππ* is reduced^40^ and hence at some geometries the order is inverted. At the optimized geometry, the S_1_^1^(*n*_Se_π_5_^*^) is dark (recall Table 1). However, the ground-state QM/MM dynamics induce slight nonplanarity, allowing the S_1_ state to gain oscillator strength. At shorter wavelengths, higher excited states contribute to the absorption spectrum and a second band peaking at 197 nm, with a shoulder around 250 nm, is obtained. The calculated spectrum agrees nicely with the experimental one,^25^ except for the intensity ratio between the low- and high-energy bands. Probably, additional electronic excited states are required to reproduce the intensity of the higher energy band. However, because we are interested in exciting 6SeGua at lower energy (see gray area in Figure 4), the agreement achieved in the lowest energy band validates the chosen QM/MM protocol.

### Nonadiabatic Dynamics

**Figure 5:**
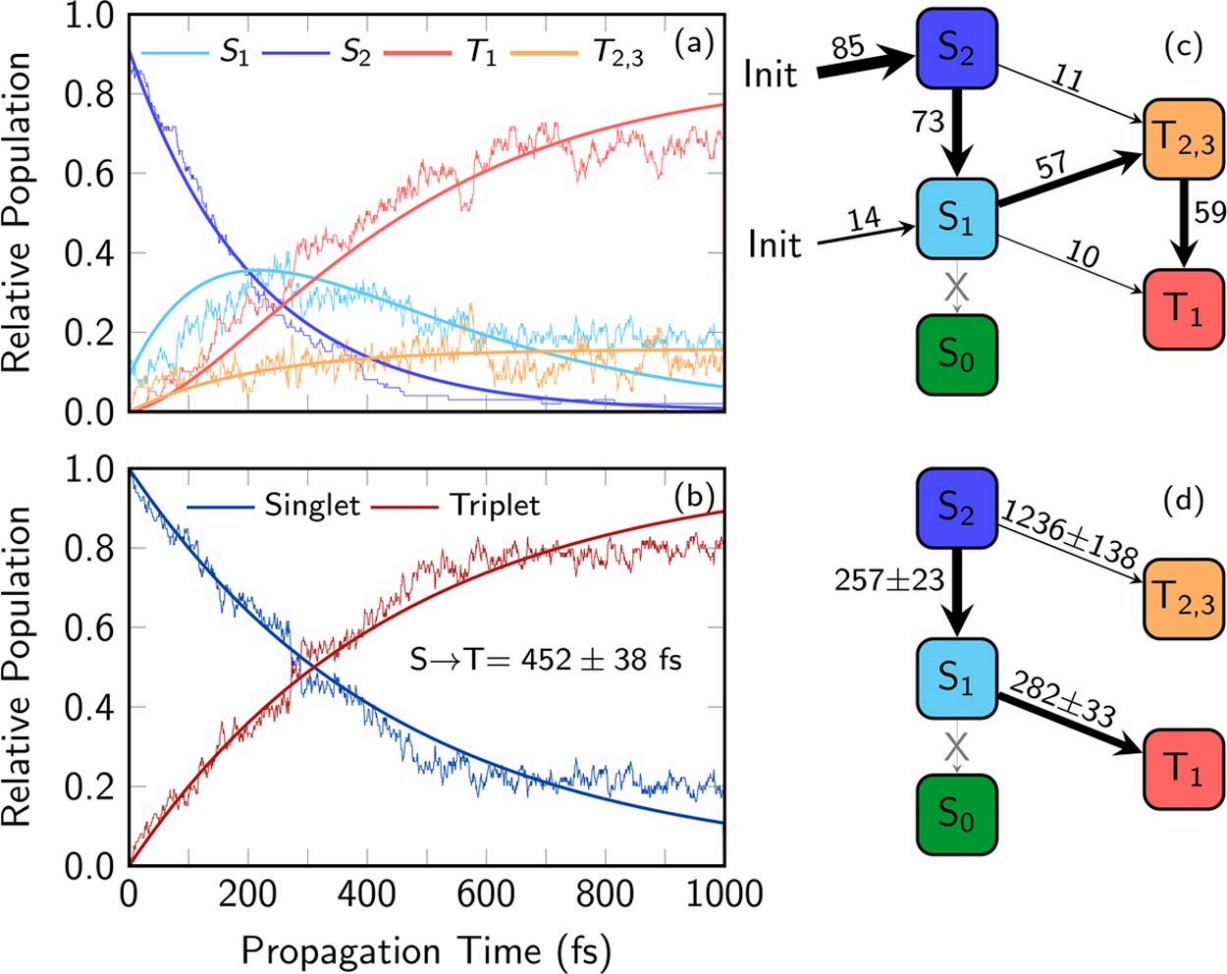
Temporal evolution of the adiabatic excited-state populations from (a) an ensemble of 99 trajectories and (b) total singlet and triplet populations. (c, d) Number of net hops and the fitted time constants, respectively. The corresponding fitted functions are plotted in panel a.

Figure 5a displays the time evolution of the electronic population of 6SeGua in water. Initially, 85% of the electronic population is excited to the S_2_ state, with the remaining 15% in the S_1_ state. After 400 fs, the S_2_ population drops to less than 10%, whereas the S_1_ and triplet-state populations increase to 25 and 68%, respectively. It is also interesting to note that the S_1_ state population initially grows and after 300 fs decreases systematically, acting as an intermediate transition en route to the triplet states. Accordingly, after 1 ps, the triplet-state population amounts to more than 80%; see Figure 5b, where the populations of singlet and triplet states are added together.

The net population transfer (difference between hops from/to state A to/from state B) among the adiabatic states is displayed in Figure 5c. The predominant relaxation pathway is clearly identified as the cascade S_2_ → S_1_ → T_2,3_ → T_1_ states (where T_2,3_ represents T_2_ and T_3_ combined, as there is only a single net hop to T_3_). It is important to realize that both the S_1_ and T_2_ states are not pure nπ* transitions along the relaxation pathway, but mixtures of ππ* and nπ* transitions, increasing their mutual SOC according to the El-Sayed rule.^89^ We also note that the system mostly obeys Kasha’s rule, as the population of the triplet states occurs predominantly from the lowest electronically excited singlet state. In addition, this deactivation pathway is in line to that found by Martínez-Fernández et al.^91^ in 6tGua using semiempirical floating numbers (FOMO–CI) scheme with SH dynamics. In 6SeGua, we also find a direct S_2_ → T_2_ path in 13% of the trajectories but no deactivation to the ground state within the simulated time.

To obtain time constants, we employed the kinetic model described elsewhere.^92^ The fitted curves are also plotted in panels a and b in Figure 5 and the corresponding time constants with errors estimated with the bootstrapping method^66,93^ are in Figure 5d. Our results suggest three phenomenological time constants for three different processes: (i) 257 fs for S_2_ → S_1_, (ii) 282 fs for S_1_ → T_1_, and (iii) 1246 fs for S_2_ → T_2,3_. The time constant regarding S_2_ → S_1_ internal conversion in the fs time scale is consistent with the calculated^40^ LIIC scans in water that bring 6SeGua directly to the (S_1_/S_2_)_CI_. We can also consider a global kinetic model with the total singlet and triplet populations (Figure 5b); this predicts an effective ISC time constant of 452 fs. It is remarkable that this is much larger than the value experimentally assigned to ISC^25^ (130 ± 50 fs) and than the one computed^91^ for the analogous 6tGua using the FOMO–CI semiempirical electronic structure method with SH dynamics (122 fs). That the FOMO–CI-SH time constant is significantly shorter than our result for 6SeGua (450 fs) is probably due to the different electronic structure method employed and the missing solvent effects. The discrepancy with the experiments will be discussed next in detail.

**Figure 6:**
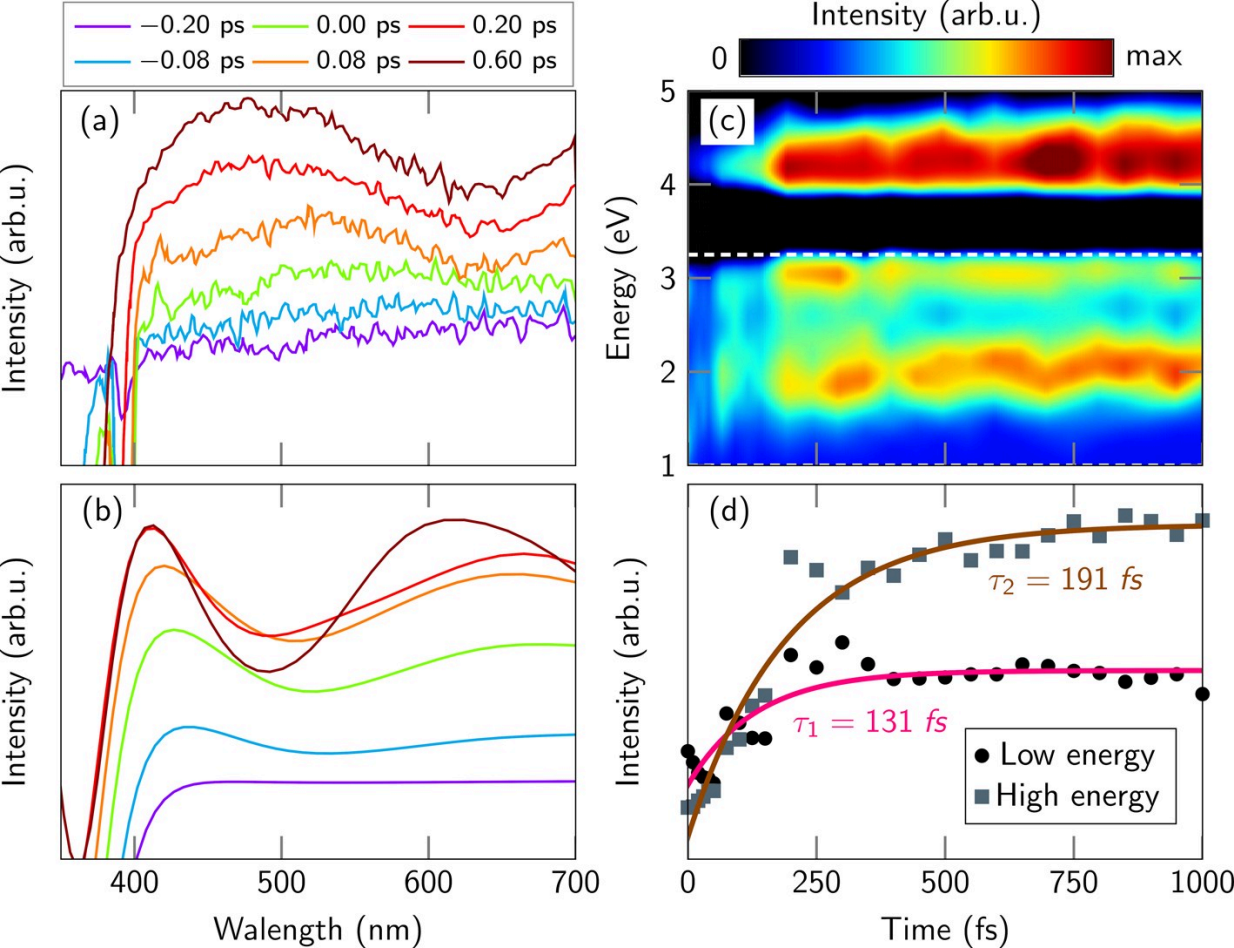
(a) Experimental TAS,^25^ (b) simulated time-convoluted TAS for some specific delay times (convoluted with a 0.25 eV × 200 fs Gaussian), (c) temporal evolution of the TAS, convoluted with a 0.25 eV Gaussian (black color includes the ground-state bleach signal), and (d) integrated simulated spectrum (without temporal convolution) together with the associated fits. The white horizontal lines in (c) define the energetic interval used to integrate the TAS. In panels b and c, the time-convoluted signals are computed as the difference between the vertical excitation energies obtained at different times and the full absorption spectrum.

Actually, to make a fair comparison with the experimental signal, it is mandatory to compute the actual observable measured in that experiment. Thus, inspired by ref (94), we computed a TAS from our trajectory data, carrying out additional vertical excitation energies for 15 singlets and 14 triplets along the trajectories. The experimental TAS^25^ at subpicosecond time delays (Figure 6a) shows a well-defined peak at 490 nm and a tail at 675 nm. Our simulated TAS (Figure 6b, c) generally reproduces the experimental intensity growth over the time, although the bands are slightly shifted in energy (e.g., the main peak is at 420 nm instead of 490 nm). We assume that this shift is due to the small basis set employed (double-ζ) and the limited capability of ADC(2) to correctly describe states with high double-excitation character, both of which lead to an inaccurate description of higher excited states. Nonetheless, it appears that ADC(2) can reproduce the presence of two absorption bands in the TAS.

To interpret the simulated TAS in Figure 6b, we performed QM/MM single-point calculations at the minima of S_2_, S_1_, T_2_, and T_1_ to obtain the ”prototypical” transient spectra of these states (Figure S4). Our results show that the experimentally observed signal (above 400 nm, 1–3 eV) is composed of transitions from S_2_^1^(ππ*), T_1_^3^(ππ*), and T_2_^3^(nπ*) to higher excited states, but with the ππ* states providing the highest intensities (peaks at 400 and 500 nm) because the T_2_ is described by a mix of ^3^(nπ*) and ^3^(ππ*) transitions. The high-energy (∼4.5 eV) band shown in Figure 6c (beyond what was probed experimentally^25^) originates from transitions from the T_1_^3^(ππ*) and S_1_^3^(nπ*) states. However, this analysis should be regarded with caution, as the molecule is in constant motion and the TAS spectra at the minima might not be fully representative. Hence, in Figure S5 we also show the decomposition of the TAS in terms of adiabatic states. At early times (until about 250 fs), the TAS is dominated by the S_2_ absorption. After this time, there is a mix of contributions coming from the other excited states, with the T_1_ having the highest contribution at late times, both in the visible and the UV ranges.

To extract time constants from our simulated TAS, we integrate the spectrum in two distinct transient absorption regions (horizontal lines in panel c: ”Low energy” between 1 and 3.25 eV; ”High energy” above 3.25 eV), omitting the temporal convolution that is included in Figure 6b, c. The integrated data (Figure 6d) is fitted monoexponentially, yielding two time constants: τ_1_ = 131 fs for the low-energy region and τ_2_ = 191 fs for the high-energy region. The former time constant is in excellent agreement with the experiment (τ_exp_ = 130 ± 50 fs),^25^ providing evidence that our calculations are reliable. This analysis shows how important is to compute the same observables as measured experimentally, if the aim is to compare with experimental values.^27^

**Figure 7:**
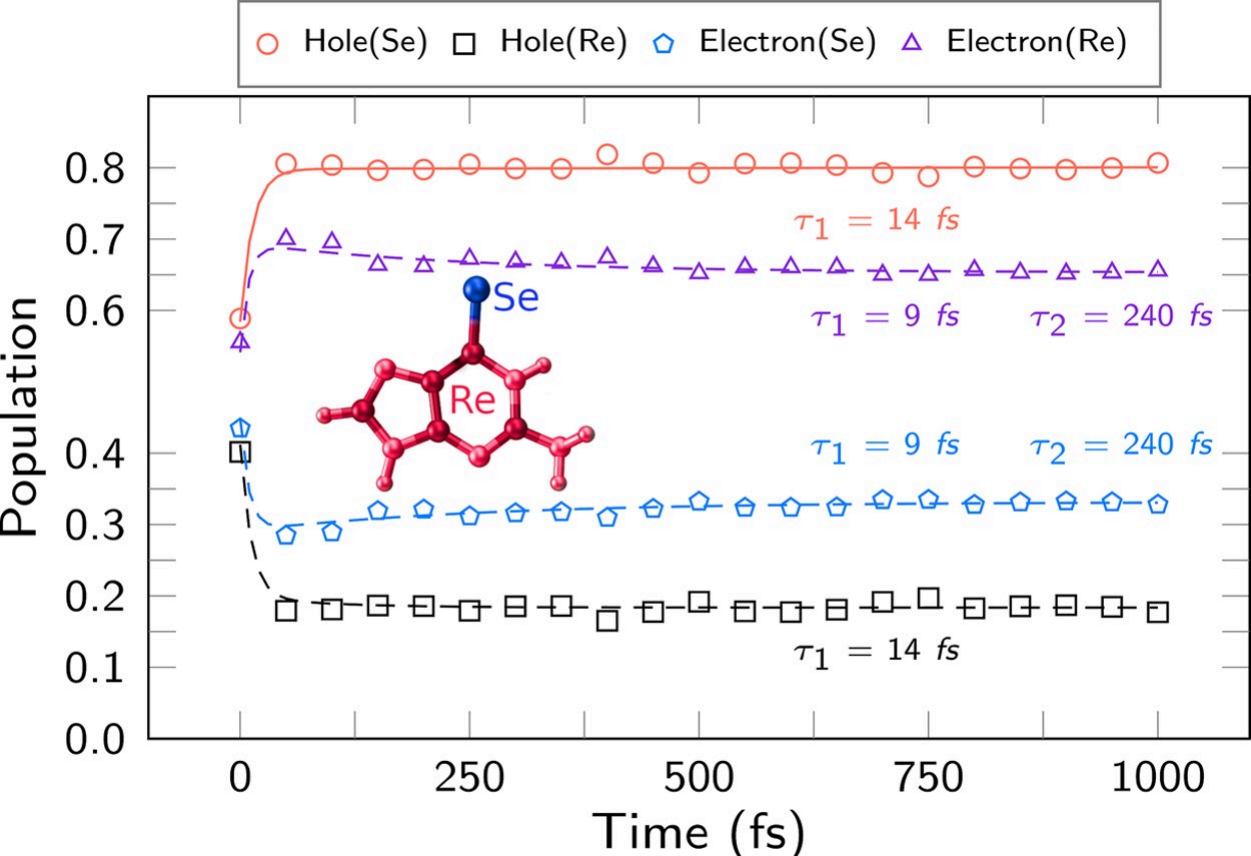
Average population of electrons and holes from 6SeGua fragmented into the Se atom (Se) and the rest (Re). Data averaged over an ensemble of 99 trajectories. Time constants are from mono- or biexponential fitting.

We now proceed to investigate the time evolution of the charge transfer (i.e., excited-state character) in 6SeGua by analyzing the one-electron transition density matrix, as implemented in the *TheoDORE* software.^86^ Two fragments were employed in this analysis: the selenium atom (labeled Se fragment) and the remaining of the molecule (Re fragment). Figure 7 displays the time evolution of the hole and electron population into each fragment, and the time constants obtained from a biexponential fitting (Table S7). Immediately after excitation, both the hole and the electron are rather delocalized over the two fragments. However, localization occurs rapidly: the hole localizes (to 80%) on Se with a time constant of 14 fs. This is likely correlated with the initial stretch of the C=Se bond (see below and Figure S6), which reduces orbital overlap and thus localizes the π_Se_ orbital more strongly on Se. The excited electron localizes within 9 fs on the Re fragment, although to a lesser extent than the hole—probably because the bond stretching modifies the localization of the π_5_^*^ and π_6_^*^ orbitals involved in the excitation. Figure 7 also shows that some electron population returns to the Se atom with a second time constant (240 fs), presumably due to the internal conversion from ππ* to nπ*.

**Figure 8:**
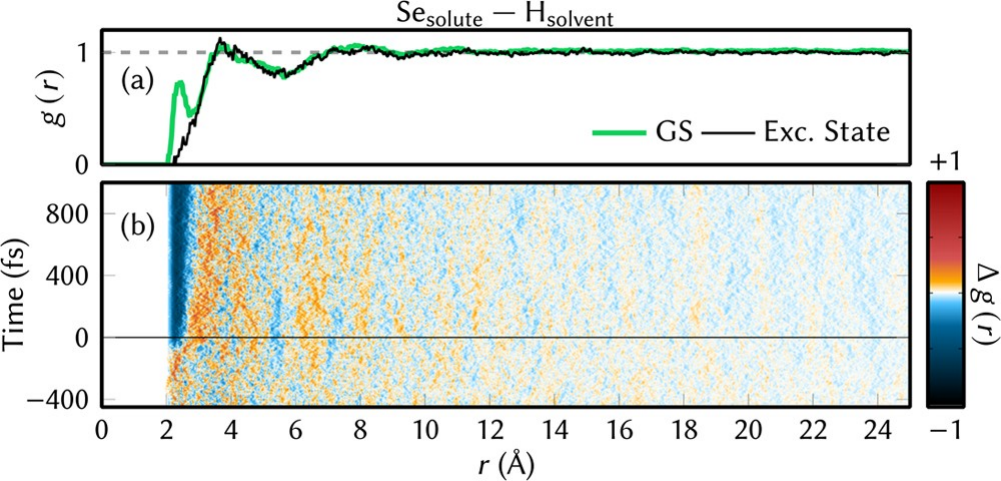
(a) Pairwise radial distribution function (*g*(*r*)) between Se_solute_ – H_solvent_ in the ground state (green line) and excited state (black line). For the excited state, an average of 99 trajectories was considered. (b) Difference between the *g*(*r*) in the excited and ground states along with the time and radial distance. Negative time corresponds to the ground state and positive to the excited state.

The charge reorganization after excitation is expected to affect the arrangement of the solvent around the solute. We investigate the solvent arrangement with the pairwise RDFs *g*(*r*) between the Se atom and the water H atoms (Figure 8). In Figure 8a, the RDF in the electronic ground state (green curve) shows a well-defined peak at 2.5 Å that suggests the formation of hydrogen bonds and thus evidence strong interaction between the Se atom and the closest water molecules. Two more solvation shells are also identified, the first one around 4 Å and the other in the 7–8 Å range. At longer distances, the RDF converges toward 1, indicating that there is no long-range solvent structure around 6SeGua.

For the electronic excited state (black line), the peak at 2.5 Å vanishes, indicating that the hydrogen bonds to Se are broken after excitation. This can be explained with the relocation of the electronic charge of 6SeGua: in any of the considered excited states, an electron is removed from a Se orbital and excited into a ring π* orbital (as discussed above). This makes the Se atom more positive, quenching its hydrogen bond acceptor capabilities. Instead, the closest water molecules very quickly reorient to form solvent–solvent hydrogen bonds. Additionally, the excited-state *g*(*r*) shows a slight increase in the second solvation shell but no changes at longer distances, as the solvent relaxation requires time to propagate outward. The temporal evolution is better displayed in Figure 8b, which shows a heatmap of the difference between current RDF and ground-state RDF. It can be seen that the hydrogen bonds are broken immediately after excitation (blue around 2 Å) and the waters driven into the second solvation shell (red around 4 Å). The same results are found in Figure S7, which shows the same difference RDFs at reduced spatial and temporal resolution to reduce noise. Note that the shown *g*(*r*) of the excited state is not representative of the steady-state solvent distribution around the excited state, and we expect further changes if the simulation time would be increased.

To capture the time scale of the solvent reorganization evident in Figure 8, we extract a slice of the time-dependent RDF at 2.5 Å (Figure S8). The associated monoexponential time constant is 129 fs, evidencing the very quick inertial solvent relaxation of water.^95^ Coincidentally, this value is very close to the experimental^25^ and simulated TAS time constants (about 130 fs), although it has a very different physical origin.

**Figure 9:**
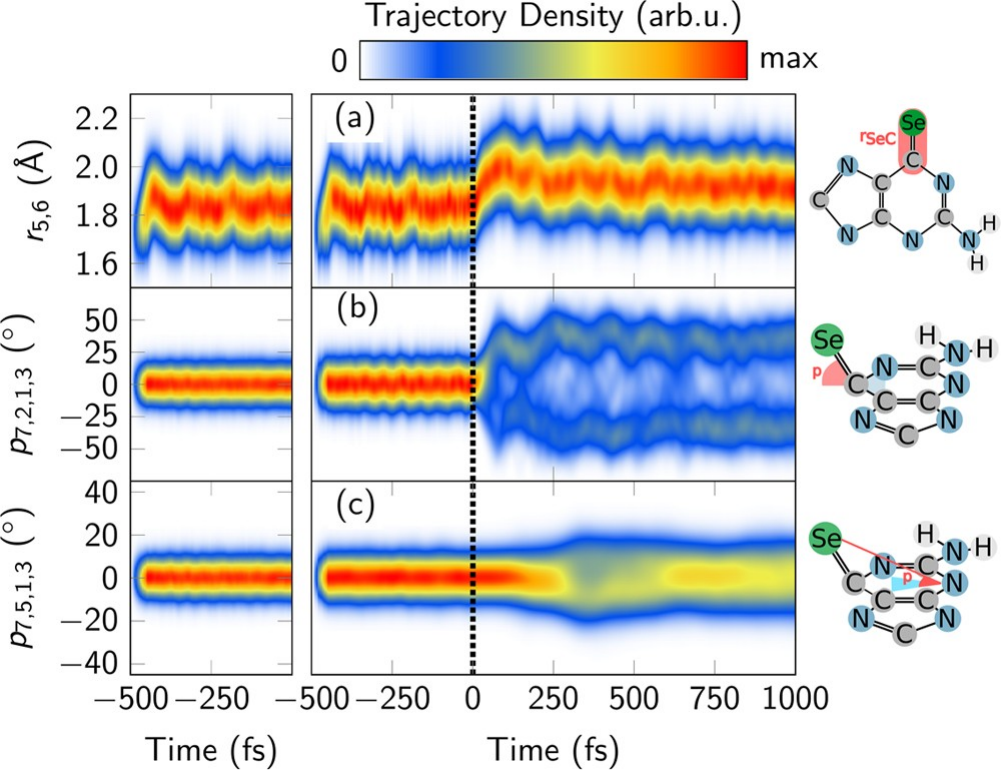
Temporal evolution of the (a) C=Se bond length, the pyramidalization angle (b) *p*_11,6,1,5_, and (c) *p*_11,3,1,5_ from an ensemble of 99 trajectories. The panels at the left side show the temporal evolution of these coordinates considering all the 500 trajectories propagated in the ground-state QM/MM dynamics, and the right panels for the 99 trajectories that are considered for the excited-state analysis.

Further mechanistic insight can be obtained by analyzing the temporal evolution of bond lengths, angles, and other geometrical parameters (Figure 9). The C=Se bond, with an average equilibrium bond length of 1.82 Å in the electronic ground state (negative times), stretches to ∼2.0 Å upon irradiation, which could correspond to the population of the S_2_ state in the early part of the dynamics. Subsequently, the bond contracts slightly and its bond length stabilizes after about 400 fs, because the S_1_, T_2_, and T_1_ minima^40^ all have similar bond lengths.

The pyramidalization angle of the Se atom (Figure 9b) is nearly zero in the electronic ground state, in agreement with its optimized value in water.^40^ Already 100 fs after excitation, most trajectories show a value of about ±40°, again in line with that observed for the optimized structures^40^ of the S_1_ (42°), S_2_ (32°), and T_1_ (40°) states and for the (S_1_/S_2_)_ISC_ crossing (24°). We notice some weak oscillations, with larger pyramidalization at around 250, 400, and 550 fs. To observe the changes in the out-of-plane displacement of the Se atom, we defined another pyramidalization angle without the C_6_.^53^ We see that this angle changes only slowly, with an oscillation period of about 700 fs. The difference between panels b and c allows us to conclude that the fast pyramidalization in panel b is due to the motion of the C_6_ atom out the plane, whereas the heavy Se atom actually stays close to the mean molecular plane. A similar trend was found in 2SeUra.^53^

To conclude this part, we collect in Table 2 all obtained time constants. As discussed in the literature,^96^ surface hopping simulations can provide two types of results, nonobservable descriptors and observables, and the obtained time constants also fall into these two categories. The S_2_ → S_1_, S_1_ → T_1_, S_2_ → T_2_, and S → T time constants are descriptors obtained from the adiabatic electronic populations. They are useful to compare with other simulations^53,91^ and facilitate generalization in terms of heuristic arguments.^96^ For example, the S → T time constant can be regarded as the overall ISC time scale of the molecule in the direct sense of a change of total spin expectation value. Additionally, the time constants derived from the charge transfer analysis should be regarded as descriptors that point to changes of the diabatic state characters. In the present case, the fast constants for charge transfer (9 and 14 fs) are related to a decrease in orbital overlap between Se and C due to the initial expansion of the Se=C bond. The slower time constant (240 fs) is possibly related to ππ* → nπ* internal conversion.

**Table 2:** Time Constants Obtained in the Present Work with Associated Processes and Source of Calculation

type	results	process	time constants (fs)
descriptor	population kinetics	S_2_ → S_1_	257 ± 23
		S_1_ → T_1_	282 ± 33
		S_2_ → T_2_	1236 ± 138
descriptor	population kinetics (ISC)	S → T	452 ± 38
descriptor	charge transfer analysis	hole migration	14
		electron migration	9 and 240
observable	simulated TAS	visible probe	131
		UV probe	191
observable	RDF between Se and H_solvent_	solvent relaxation	129
observable	C=Se bond length	nuclear relaxation	14 and 123

The descriptor time constants should be contrasted with the time constants associated with observables—the latter are the values needed for a correct comparison with experimental time constants.^94,97,98^ The experimental TAS time constant^25^ of 130 fs fits excellently with the simulated TAS time constant (131 fs) obtained for the visible probe range but is in stark contrast to the ISC time scale of about 450 fs from the populations. This clearly shows that the experimental time scale not (only) measures ISC but is an effective time constant that includes multiple processes, such as leaving the Franck–Condon region, internal conversion from ππ* to nπ*, ISC, or solvent relaxation. The disparity between both time constants (130 and 450 fs) is a neat example of the differences between the actual observable signal (TAS) and idealized underlying independent processes (e.g., ISC) only directly accessible using simulations.

Our simulations also predict additional time constants that might be observed by additional suitable experiments. The second time constant from TAS, 191 fs, could be (in principle) observable by TAS employing an UV probe laser, although in practice such measurement is probably difficult because the ground state absorbs in the same UV region. The time constant of solvent reorganization (129 fs) obtained from the RDFs could be observed (together with all other pair RDFs) through fs-resolved X-ray scattering experiments.^99^ Even though this time constant almost perfectly matches the first TAS time constant, it has a different physical origin, arising from the reorganization properties of the solvent.^95^ In a similar way, scattering experiments can measure intramolecular bond length distributions (see, e.g., ref (100)), and hence the time constants of C=Se bond stretch (14 and 123 fs) are in principle experimentally accessible.

### Lifetime of the Triplet State

**Figure 10:**
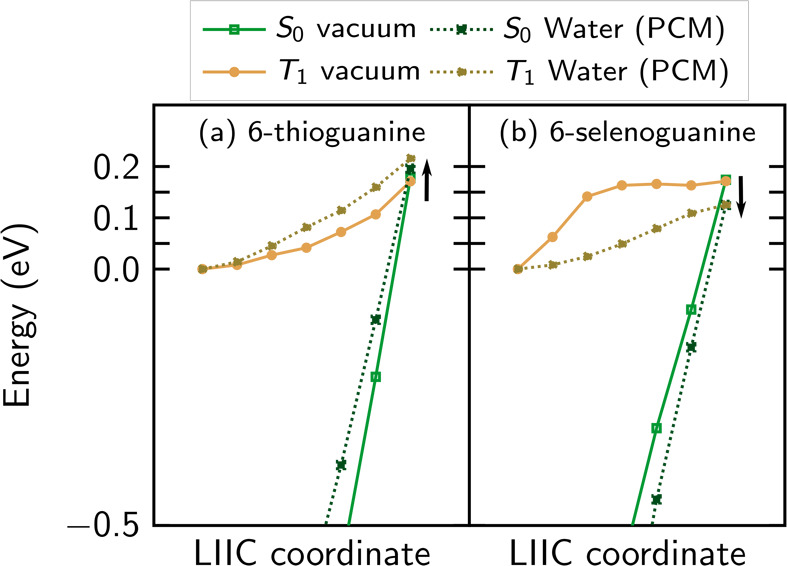
LIIC paths between the (T_1_)_min_ and (T_1_/S_0_)_ISC_ structures for (a) 6tGua and (b) 6SeGua in a vacuum and in water. For each structure generated from the LIIC method, an energy calculation was performed at the MS-CASPT2. Black arrows indicate how the barrier changes when solvation effects are added.

Lastly, we address the interesting finding that the triplet state of 6SeGua is very short lived (1.7 ns), and thus experimentally measured to be 835 times shorter than the triplet lifetime in 6tGua (1420 ns) in aqueous solution.^25^ As such long nonadiabatic simulations are unfeasible and the SOC between the ground state and the triplet states is not available in the ADC(2) implementation we are using,^59^ we optimize the (T_1_)_min_ and (T_1_/S_0_)_ISC_ geometries for both 6tGua and 6SeGua and compute the relevant LIIC pathway in vacuum and water (Figure 10.). These calculations are done at the MS-CASPT2(12,10)/cc-pVDZ level of theory with implicit solvent via the polarizable continuum model.^101^

The energy barrier in a vacuum is the same for both molecules (0.17 eV, solid lines), but the barrier in water increases by 0.05 eV in 6tGua (0.22 eV) and decreases by approximately the same amount in 6SeGua (0.12 eV). The latter barrier can be compared to the values of 0.26 eV in water and 0.30 eV in DNA, computed through less accurate CASSCF/MM optimizations.^40^ Our results indicate that in 6tGua, the pathway to the ground state is more difficult than for 6SeGua and, consequently, the electronic population will be trapped longer in the 6tGua triplet state than in 6SeGua, thereby increasing its triplet lifetime. A second reason for the long triplet lifetime in 6tGua could be the presence of a second triplet minimum,^102^ missing in 6SeGua. Third, it can be assumed that the significantly larger SOCs in 6SeGua also contribute to its short triplet life times. Similar trends for barrier heights, minima, and SOCs were previously found for 2SeUra,^53^ which suggests that short triplet life times could be a general property of selenobases.

## Conclusions

In conclusion, QM/MM nonadiabatic dynamics simulations and complementary static calculations allowed us to clarify the relaxation pathways after irradiating 6SeGua in water. After excitation of the S_2_ bright state, the first photochemical event is an ultrafast internal conversion to the S_1_ excited states. After that, the T_2_ triplet state is populated by intersystem crossing and finally, the lowest T_1_ state is reached by internal conversion within the triplet manifold. This main relaxation mechanism complies with conventional wisdom, as triplet states are typically accessed from the lowest singlet excited state. The short lifetime of the T_1_ state compared to that of its thiobase analogue 6tGua can be rationalized by the absence of a second minimum on the T_1_ potential, a decrease in the relevant activation barrier, and the increased SOCs in 6SeGua. The analysis of the nuclear motion of solute and solvent points to the role of the carbon linked to the Se, and how charge migration from the Se atom to the rest of the molecule controls solvent relaxation.

Another important finding of our work is derived from the simulated transient absorption spectra that provide two time constants, 131 and 191 fs, the former in excellent agreement with the experimental^25^ value (130 fs). Our simulations clearly illustrate that the experimental time constant of 130 fs is not purely ISC—which we predict to have a time constant of 450 fs—but a complex mixture of different overlapping processes. In addition to the TAS analysis, other time constants were also obtained and correlated with specific processes that are taking place simultaneously on this time scale. Some of these time constants can be related to different time-resolved measurements techniques.

This work therefore emphasizes the importance of calculating the actual experimental observables for fair comparisons and the power of theoretical calculations to disentangle the complexity behind experimental signals.

## Supporting Information Available

Further stationary calculations, SOC contribution to the electronic absorption spectrum, validation of the level of theory, further nonadiabatic dynamics results, and Cartesian coordinates of the optimized structures in XYZ format (PDF)
