# Supplementary material for: Ultrafast Intersystem Crossing Dynamics of 6-Selenoguanine in Water
Source: JACS Au. 2022 Jun 28;2(7):1699–711. doi: 10.1021/jacsau.2c00250 (PMC9327080; doi:10.1021/jacsau.2c00250)
Supplement: Supplementary file 1 — au2c00250_si_001.pdf [file au2c00250_si_001.pdf]

Supporting Information:

Ultrafast Intersystem Crossing Dynamics of 6-Selenoguanine in Water

Danillo Valverde†,‡,§, Sebastian Mai¶, Sylvio Canuto‡, Antonio Carlos Borin†, Leticia González*,¶

†*Department of Fundamental Chemistry, Institute of Chemistry, University of São Paulo, Avenida Professor Lineu Prestes, 748, São Paulo, SP, CEP 05508-000, Brazil*

‡*Institute of Physics, University of São Paulo, São Paulo, SP, 05508-090, Brazil*

¶*Institute of Theoretical Chemistry, Faculty of Chemistry, University of Vienna, Vienna, Austria*

§*Now at Unité de Chimie Physique Théorique et Sctructurale, Namur Institute of Structured Matter, Université de Namur, B-5000 Namur, Belgium*

**Email: leticia.gonzalez@univie.ac.at*

Table of Contents

Table of Contents 1

Section S1. Further Stationary Calculations 2

Section S2. SOC contribution in the electronic absorption spectrum 5

Section S3. Validation of the level of theory 5

Subsection S3.1. Vertical excitation energies 5

Subsection S3.2. Excited-state minima and crossing points 6

Subsection S3.3. Excited-state deactivation pathways 9

Section S4. Further Dynamical Results 11

Section S5. Optimized Geometries 14

References 17

Section S1. Further Stationary Calculations

Table S1: Bond lengths (Å) for the ground state of 6SeGua in gas phase computed at the MP2 level using the cc-pVDZ and ANO-RCC-VDZP basis sets. The Douglas-Kroll-Hess formalism is included to account for scalar-relativistic effects when the ANO-RCC-VDZP basis set is considered.

Bond Length	cc-pVDZ	ANO-RCC-VDZP
r(N_1C_2)	1.371	1.375
r(C_2C_3)	1.315	1.322
r(N_3C_4)	1.356	1.362
r(C_4C_5)	1.403	1.410
r(C_5C_6)	1.424	1.432
r(N_1C_6)	1.404	1.406
r(C_2N_{10})	1.364	1.365
r(C_2Se_{11})	1.801	1.799

Table S2: Vertical excitation energies (*ΔE*, in eV) and associated oscillator strengths (*f*) of isolated 6SeGua computed at the MS(4S,3T)-CASPT2(14,10) level using the cc-pVDZ, ANO-RCC-VDZP, and cc-pVDZ-DK basis sets. The Douglas-Kroll-Hess formalism was used only for the latter two basis sets. These calculations were performed at the MP2/ANO-RCC-VDZP ground state optimized structure.

Nature	cc-pVDZ		ANO-RCC-VDZP		cc-pVDZ-DK	
	ΔE	*f*	*ΔE*	*f*	*ΔE*	*f*
S_1 $^1(n_{Se}\pi_5^*)$	2.76	0.000	2.76	0.000	2.64	0.000
S_2 $^1(\pi_{Se}\pi_5^*)$	3.45	0.472	3.38	0.343	3.59	0.338
S_3 $^1(\pi_{Se}\pi_6^*)$	4.34	0.054	4.63	0.044	4.30	0.049
T_1 $^3(\pi_{Se}\pi_5^*)$	2.43		2.46		2.51	
T_2 $^3(n_{Se}\pi_5^*)$	2.66		2.70		2.58	
T_3 $^3(\pi_{Se}\pi_6^*)$	3.85		3.88		3.75	

Table S3: Spin-orbit coupling (SOC, in cm^{-1}) elements between different singlet and triplet states for 6SeGua in gas phase. These calculations were performed at the MS(4S,3T)-CASPT2(14,10) and ADC(2) levels with different basis sets. The Douglas-Kroll-Hess formalism was used for the ANO-RCC-VDZP and cc-pVDZ-DK basis sets. These calculations were performed at the MP2/ANO-RCC-VDZP ground state optimized structure.

	MS-CASPT2			ADC(2)
SOC	cc-pVDZ	cc-pVDZ-DK	ANO-RCC-VDZP	cc-pVDZ
$S_1 \rightarrow T_1$	532.2	551.5	552.9	357.6
$S_1 \rightarrow T_2$	9.1	12.0	4.6	33.7
$S_2 \rightarrow T_1$	6.4	13.2	11.7	30.6
$S_2 \rightarrow T_2$	354.4	385.2	382.9	336.7

Table S4: Vertical excitation energies (in eV) and corresponding oscillator strengths (f) for 9 singlet and 6 triplet states of 6SeGua in gas phase computed at the ADC(2)/cc-pVDZ level.

State character	cc-pVDZ	
S_1 $^1(n_{Se}\pi_5^*)$	2.65	0.000
S_2 $^1(\pi_{Se}\pi_5^*)$	3.45	0.380
S_3 $^1(\pi_{Se}\pi_6^*)$	4.47	0.080
S_4 $^1(n_{Se}\pi_6^*)$	4.49	0.002
S_5 $^1(\pi_4\pi_5^*)$	5.35	0.017
S_6 $^1(n_N\pi_6^*)$	5.48	0.000
S_7 $^1(\pi_3\pi_5^*)$	5.91	0.018
S_8 $^1(n_N\pi_5^*)$	5.99	0.006
T_1 $^3(\pi_{Se}\pi_5^*)$	2.51	
T_2 $^3(n_{Se}\pi_5^*)$	2.54	
T_3 $^3(\pi_{Se}\pi_6^*)$	4.04	
T_4 $^3(n_{Se}\pi_6^*)$	4.48	
T_5 $^3(\pi_4\pi_5^*)$	4.53	
T_6 $^3(n_N\pi_5^*)$	5.24	

Table S5: Atomic charges for 6SeGua obtained by using the RESP procedure at the B3LYP/cc-pVDZ level. Atomic labels: N = nitrogen (blue), C = carbon (gray), Se= selenium (green), H = hydrogen (white).

Site	Charges
N1	-0.0758
C2	0.5635
N3	-0.6085
C4	0.3995
C5	0.5326
C6	-0.3198
N7	-0.5990
C8	0.1731
N9	-0.5147
N10	-0.8043
Se11	-0.3055
H12	0.2308
H13	0.3745
H14	0.3745
H15	0.3986
H16	0.1804

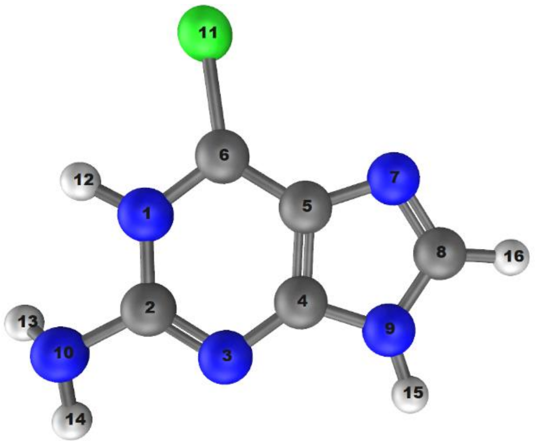

Listing S1: Force field parameters used for 6SeG in order to model the molecular interaction in the classical molecular dynamics simulation. The parameters are given in frcmod format for use in AMBER.

```
# BOND
 se-c2    500 1.730 article 2SeThy J. Phys. B 2011, 115, 10041

# ANGLE
 na-c2-se 100.000 122.860  article 2SeThy J. Phys. Chem. B 2011, 115, 10041
 c2-c2-se 100.000 124.080  article 2SeThy J. Phys. Chem. B 2011, 115, 10041
 na-c2-nh  73.100 109.330  same as na-c2-na, penalty score=  3.0

# Dihedrals Improper
 c2-na-c2-se 1.1 180.0 2.0 Using the default value
 c2-c2-na-hn 1.1 180.0 2.0 General improper torsional angle X- X-na-hn
 n2-na-c2-nh 1.1 180.0 2.0 Using the default value
 c2-hn-nh-hn 1.1 180.0 2.0 Same as X -X -na-hn, penalty score= 41.2
 c2-n2-c2-na 1.1 180.0 2.0 Using the default value
 h5-n2-c2-na 1.1 180.0 2.0 Using the default value
 c2-c2-c2-n2 1.1 180.0 2.0 Using the default value

# Nonbonded parameters
 se          2.075  0.585 article 2SeThy  J. Phys. Chem. B 2011, 115, 10041
```

Section S2. SOC Contribution to the Electronic Absorption Spectrum

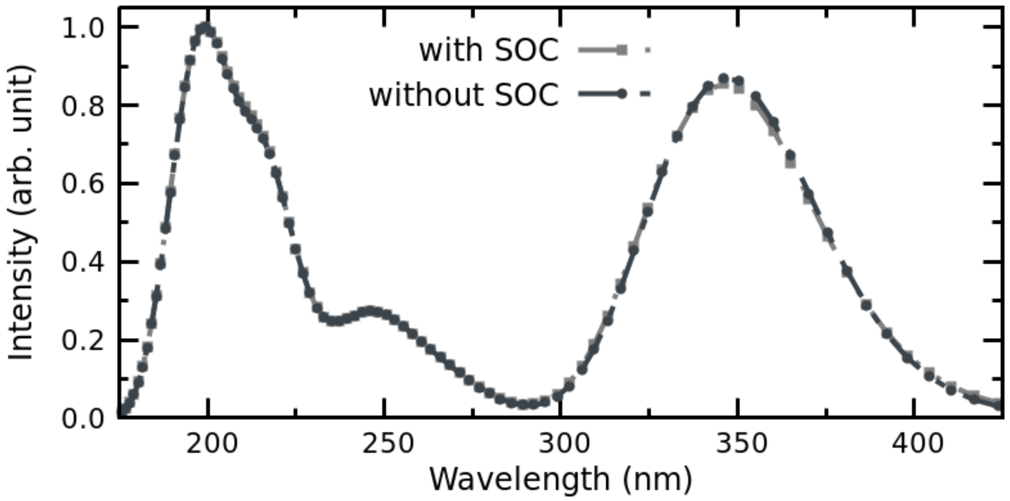

Figure S1: Electronic absorption spectrum of 6SeGua in water with/without SOC contribution. This spectrum is emulated by sampling 500 snapshots from the QM/MM MD in the ground state. At each snapshot, a total of 15 singlets and 14 triplets states were computed at the ADC(2)/cc-pVDZ level.

Section S3. Validation of the Level of Theory

Here we report a gas-phase comparison between ADC(2) and MS-CASPT2 vertical excitation energies, critical points and deactivation pathways as obtained from LIIC scans.

Subsection S3.1. Vertical Excitation Energies

6SeGua gas phase vertical excitation energies at both levels of theory are displayed in Table 1 of the main manuscript. All methods predict a low-lying $^1(n_{Se}\pi_5^*)$ excited state (the S_1 at the equilibrium geometry), with similar energies computed with ADC(2) (2.76 eV), MS-CASPT2(14,12) (2.76 eV) , and MS-CASPT2(12,10) (2.83 eV) methods. This $^1(n_{Se}\pi_5^*)$ state consist of a single electronic excitation from the lone pair localized on the Se atom (n_{Se}) to the π_5 antibonding orbital. This state is similar in 2-selenouracil (2SeUra) $^1(n_{Se}\pi_2^*)$ [S1], although in the 6SeGua it is at the MS-CASPT2 level 0.5 eV lower in energy. As for 2SeUra, the S_1 $^1(n_{Se}\pi_5^*)$ electronic state is dark and does not contribute to the absorption spectrum.

The S_2 state at the Franck-Condon (FC) region is the $^1(\pi_{Se}\pi_5^*)$, computed at 3.45 eV with the best MS-CASPT2(14,12) method, in agreement with the experimental value [S2]. The excitation energies computed with ADC(2) (3.61 eV) and TD-B3LYP (3.60 eV) agree better with that computed at the MS-CASPT2(12,10)/cc-pVDZ level of theory, that is, with a smaller active space. This state is associated with the highest oscillator strength, which confirms the S_2 $^1(\pi_{Se}\pi_5^*)$ as the bright state.

The S_3 is a $^1(\pi_{Se}\pi_6^*)$ state, computed at 4.64 eV with MS-CASPT2(12,10) and ADC(2) levels of theory, and 4.34 eV at the MS-CASPT2(14,12) level. The associated oscillator strength is small (~ 0.05). Given its energetic separation with the lower-lying states and small oscillator strength, its contribution to the relaxation mechanisms after excitation to the first absorption band can be neglected.

The two lowest triplet states are the T_1 $^3(\pi_{Se}\pi_5^*)$ and T_2 $^3(n_{Se}\pi_5^*)$ states. All levels of theory predict the triplet states in the same energetic region (T_1: 2.4 – 2.6 eV; T_2: 2.6 – 2.9 eV). Both triplet states are also excitations from the selenium orbitals, as observed for the singlet states. Excitations from other orbitals give rise to triplet states with a larger energy gap (≥ 3.8 eV) (see Table S_3), so that we can focus on two lowest triplet states. The same pattern is observed for 2SeUra [S1].

Summarizing the performance of the three levels of theory in describing the 6SeGua lowest lying excited states: the energetic order of the excited state is the same and the relative energies in the FC region agree. The S_2 state is ~ 0.9 eV above the S_1 state at the MS-CASPT2(12,10)/cc-pVDZ and ADC(2)/cc-pVDZ levels of theory, being more stabilized at the MS-CASPT2(14,12)/cc-pVDZ level; the S_3 state is computed to be at about 0.9 – 1.0 eV higher than the S_2 state in the three cases; the T_2 state is placed a bit closer in energy to the T_1 state (0.08 eV) at the ADC(2)/cc-pVDZ level of theory, while the gap between the triplet states computed with the MS-CASPT2 method is larger (0.28 – 0.24 eV).

Subsection S3.2. Excited-State Minima and Crossing Points

Gas phase electronic states geometries and minimum energy crossing points were optimized at the MS-CASPT2(12,10)/cc-pVDZ and ADC(2)/cc-pVDZ levels of theory. Representative

optimized structures are displayed in Figure S2 and relevant conformational parameters and relative energies are collected in Table S6. The optimized structures were classified according to the six-membered ring parameters proposed by Cremer-Pople [S3] (Q, amplitude) and Boeyens [S4] for ring conformations (envelope (E), boat (B), screw-boat (S), twist-boat (T), half-chair (H), etc). The indices in the symbols represent the atoms moving out of the ring plane.

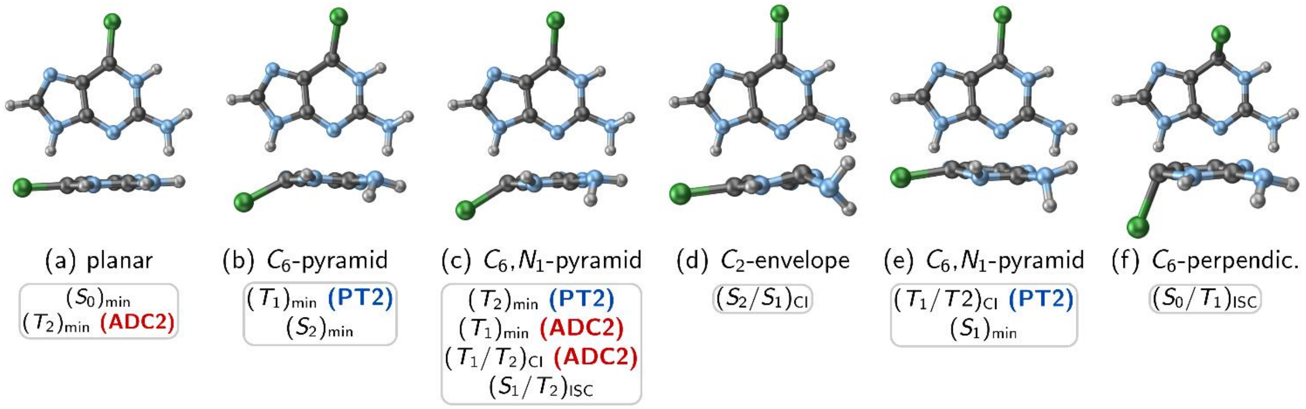

Figure S2: 6SeGua gas phase optimized geometries and minima energy crossing points computed at ADC(2)/cc-pVDZ and MS-CASPT2(12,10)/cc-pVDZ levels of theory. The geometries were gathered according to Cremer-Pople and Boeyens parameters, The “ADC2” and “PT2” labels indicate that a given critical point was classified differently depending on the level of theory.

The optimized ground state geometry is nearly planar at both levels of theory, except for the hydrogen atoms of the amino group, which are outside the molecular plane by 48°. The first singlet excited state S_1 $^1(n_{Se}\pi_5^*)$ has a minimum located adiabatically around 2.5 eV above the ground state minimum. In both levels, the optimized structure is classified to be in the same Boeyens group ($^1H_6$), but the pyramidalization on the selenium (MS-CASPT2: 6.7° and ADC(2): 24.2°) and H_{12} (MS-CASPT2: 18.5° and ADC(2): 25.5°) atoms is slightly different.

The minimum on the S_2 $^1(\pi_{Se}\pi_5^*)$ PES is placed adiabatically 3.0 eV above the ground state. The most important difference with respect to the ground state minimum is the elongated C=Se bound (~0.2 Å) and the pyramidalization of the C_6 atom, moving the selenium atom out of plane by about 50° at the MS-CASPT2 (42° degree at ADC(2)).

For the T_1 $^3(\pi_{Se}\pi_5^*)$ minimum, the optimized structures resemble much each other at both levels of theory. The largest discrepancy is observed for the T_2 $^3(n_{Se}\pi_5^*)$, for which the six-membered ring itself is planar with the selenium (~ 32°) and H_{12} atoms (~ 19°) out of the plan,

while the ADC(2) corresponding optimized geometry is fully planar.

Table S6: Energies (E, in eV), bond lengths (A), angles (°), Cremer-Pople (Q, A) parameter and Boeyens classification for the minimum energy crossing points of 6SeGua at the MS-CASPT2(12,10)/cc-pVDZ and ADC(2)/cc-pVDZ levels.

Minima										
	S_0 $^1(gs)_{min}$		S_1 $^1(n_{Se}\pi_5^*)_{min}$		S_2 $^1(\pi_{Se}\pi_5^*)_{min}$		T_1 $^3(\pi_{Se}\pi_5^*)_{min}$		T_2 $^3(n_{Se}\pi_5^*)_{min}$	
Parameters	PT2	ADC2	PT2	ADC2	PT2	ADC2	PT2	ADC2	PT2	ADC2
E	0.0	0.0	2.45	2.50	3.03	2.95	2.25	2.30	2.38	2.44
$r(N_1C_2)$	1.373	1.374	1.376	1.386	1.363	1.367	1.349	1.377	1.376	1.382
$r(C_2N_3)$	1.319	1.319	1.327	1.310	1.332	1.326	1.343	1.316	1.319	1.315
$r(N_3C_4)$	1.372	1.364	1.376	1.385	1.352	1.363	1.364	1.381	1.380	1.385
$r(C_4C_5)$	1.407	1.409	1.408	1.400	1.423	1.420	1.423	1.402	1.404	1.401
$r(C_5C_6)$	1.430	1.435	1.421	1.430	1.426	1.430	1.434	1.444	1.431	1.422
$r(N_1C_6)$	1.416	1.405	1.432	1.430	1.409	1.420	1.442	1.433	1.429	1.428
$r(C_2N_{10})$	1.385	1.389	1.368	1.399	1.384	1.391	1.389	1.398	1.396	1.372
$r(C_2Se_{11})$	1.820	1.793	1.922	1.914	2.073	1.997	1.920	1.911	1.932	1.906
$p(Se_{11}C_2N_1C_5)$	1.0	0.5	27.3	24.2	49.2	42.2	37.7	34.0	31.8	0.0
$p(H_{12}N_1C_2C_6)$	7.0	2.5	18.5	25.5	5.7	3.3	1.4	17.3	18.9	0.0
Q	0.0	0.0	0.09	0.06	0.02	0.06	0.07	0.04	0.04	0.00
Boyens	–	–	$^1H_6$	$^1H_6$	$^5H_6$	E_6	E_6	$^1H_6$	$^5H_6$	–

Minimum Energy Crossing Points									
	$(S_1/S_2)_{CI}$ $^1(n_{Se}\pi_5^*)/^1(\pi_{Se}\pi_5^*)$		$(S_1/T_2)_{ISC}$ $^1(n_{Se}\pi_5^*)/^3(n_{Se}\pi_5^*)$		$(T_1/T_2)_{CI}$ $^3(\pi_{Se}\pi_5^*)/^3(n_{Se}\pi_5^*)$		$(T_1/S_0)_{ISC}$ $^3(\pi_{Se}\pi_5^*)/^1(gs)$		
	PT2	ADC2	PT2	ADC2	PT2	ADC2	PT2	ADC2	
E	3.20	3.17	2.46	2.63	2.50	2.34	2.43	2.52	
$r(N_1C_2)$	1.442	1.433	1.386	1.392	1.383	1.368	1.348	1.363	
$r(C_2N_3)$	1.407	1.446	1.308	1.308	1.301	1.321	1.343	1.329	
$r(N_3C_4)$	1.303	1.290	1.388	1.387	1.381	1.375	1.348	1.365	
$r(C_4C_5)$	1.449	1.488	1.398	1.399	1.393	1.409	1.432	1.418	
$r(C_5C_6)$	1.380	1.372	1.431	1.427	1.421	1.443	1.465	1.468	
$r(N_1C_6)$	1.356	1.364	1.428	1.430	1.427	1.435	1.450	1.448	
$r(C_2N_{10})$	1.427	1.429	1.398	1.399	1.413	1.397	1.376	1.390	
$r(C_2Se_{11})$	1.908	1.889	1.934	1.915	1.915	1.912	1.955	1.917	
$p(Se_{11}C_2N_1C_5)$	0.8	2.2	28.2	21.6	19.1	34.8	59.5	55.5	
$p(H_{12}N_1C_2C_6)$	11.7	3.4	22.6	29.4	32.5	9.6	0.4	2.3	
Q	0.16	0.15	0.04	0.06	0.110	0.03	0.07	0.10	
Boyens	^2E	$^{2,5}B$	$^1H_6$	$^1H_6$	$^1H_6$	$^5H_6$	E_6	$^1S_6$	

Besides minima, we also optimized minimum energy crossing points (MECP) with both levels of theory. The search for a MECP between the S_1 and S_2 states indicate that at this point there is a three-state near-degeneracy encompassing the S_1, S_2, and T_2 states; nonetheless, we still label this point as $(S_1/S_2)_{CI}$, because in this region the nonadiabatic couplings between the S_1 and S_2 states are likely to be more relevant for the photophysics that the singlet-triplet couplings as we will comment below. Thus, we have not explicitly optimized a three-state degenerate structure. At both levels, this $(S_1/S_2)_{CI}$ geometry is

classified as envelope at C_2 position (E_2), with the amino group hydrogen atoms almost perpendicular to the molecular plane.

A conical intersection between the two lowest lying triplet states was also optimized and shows a pyramidalization of the C_6 atoms of about 19° at MS-CASPT2 and 35° at ADC(2). We have also found two singlet-triplet crossing points between the S_1 and T_2 states ($(S_2/T_2)_{ISC}$), and between the lowest triplet state $^3(\pi_{Se}\pi_5^*)$ and the ground state ($(T_1/S_0)_{ISC}$). The $(S_1/T_2)_{ISC}$ structure resembles the $(S_1)_{min}$ geometry, whereas the $(T_1/S_0)_{ISC}$ structure presents the largest pyramidalization angle of the selenium atom among all optimized geometries.

Subsection S3.3. Excited-State Deactivation Pathways

Here we investigate the gas phase photochemical pathways using with the linear interpolation in internal coordinates (LIIC) methodology. Note that MS-CASPT2 scans are smooth and do not exhibit the "wiggle" artifacts sometimes reported for MS-CASPT2 calculations [S6].

Figure S3 depicts the evolution of the GS, S_1, S_2, T_1, and T_2 states along the LIIC photochemical pathways among different regions of their PESs, computed at ADC(2)/cc-pVDZ (panel (a)) and MS-CASPT2(14,12)//MS-CASPT2 (12,10)/cc-pVDZ (panel b) levels of theory.

PATH I connects the FC and S_2 $^1(\pi_{Se}\pi_5^*)_{min}$ regions. As it can be noticed, the S_2 state evolves barrierless from the FC region to its minimum. The next step goes from the $(S_2)_{min}$ to the $(S_1/S_2)_{CI}$ region, on which one observes an energetic barrier computed at the MS-CASPT2/cc-pVDZ level to be about 0.41 eV (11.7 kcal/mol), a little bit lower than that computed with the ADC(2) method (0.62 eV; 14.2 kcal/mol). In a recent work [S5] the barrier was estimated to be ~10.2 kcal/mol with MS-CASPT2 (using geometries optimized at CASSCF level). It is noteworthy that this barrier is completely absent in water [S7], which will lead to a faster population transfer to the S_1 state in solution.

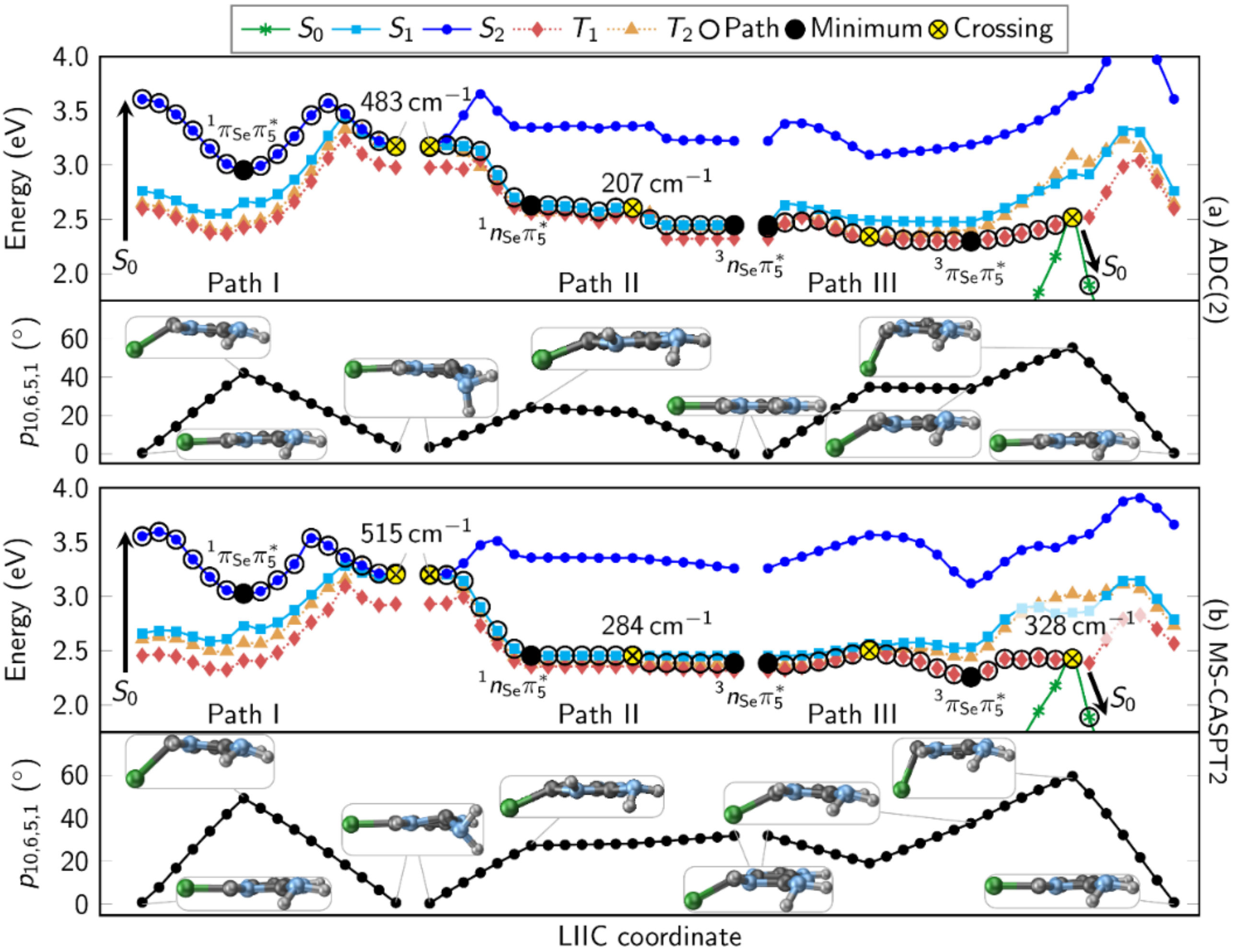

Figure S3: 6SeGua gas phase linear interpolation in internal coordinates pathways connecting optimized geometries and minimum energy crossing points computed at (a) ADC(2)/cc-pVDZ and (b) MS-CASPT2(14,12)/cc-pVDZ//MS-CASPT2(12,10)/cc-pVDZ levels of theory. The relaxation paths are indicated with open black circles, minima with black circles and crossing points with X. The black arrows show the vertical excitation from the FC region to the second singlet excited state. SOCs between crossing points related to ISC are shown in cm^{-1}.

The next photochemical step, labelled PATH II, connects the (three-state) near-denegeracy $(S_1/S_2)_{CI}$ and the $(S_1)_{min}$ structures. This minimum is reached from the $(S_1/S_2)_{CI}$ region by an energy of 0.75 eV and 0.67 eV with MS-CASPT2 and ADC(2) methods, respectively. In the vicinity of this minimum, the triplet state may be easily accessed by a crossing between the S_1 and T_2 states. This crossing point is a bit higher in comparison with the $(S_1)_{min}$ (0.01 eV with MS-CASPT2 and 0.13 eV with ADC(2)). Both methods predict a smaller SOC on this region in relation to the $(S_1/S_2)_{CI}$ crossing point (284, and 207 cm^{-1} with

MS-CASPT2 and ADC(2) methods, respectively). Note that there is a mix of contributions coming from $n\pi^*$ and $\pi\pi^*$ transitions at the crossing regions, enabling a large SOC. From here, the system may follow towards the minimum of the T_2 state.

The last path (PATH III) starts on the $(T_2)_{min}$, from which the system can evolve towards a conical intersection with the T_1 state. Subsequently, the system is lead to a minimum on the T_1 hypersurface releasing an energy of 0.03 eV with ADC(2) and 0.24 eV with MS-CASPT2. As some point in time, the system is expected to return to the ground state.

Section S4. Further Nonadiabatic Dynamics Results

Table S7: Coefficients obtained from a biexpoential fit (f(t)=A*exp(-t/τ1)+B*exp(-t/τ2)+C) of the hole (electron) population for the two fragments considered.

	A	B	C	τ_1	τ_2
Hole(Se)	-0.214	-0.487	1.286	14	225781
Hole(Re)	0.213	0.016	0.183	14	9
Electron(Se)	0.158	-0.041	0.331	9	256
Electron(Re)	-0.154	0.042	0.653	9	234

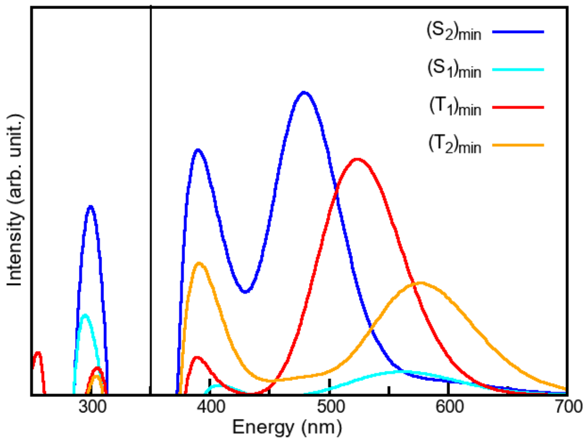

Figure S4: TAS spectrum simulated based on the vertical excitation energies calculations at the excited-state minima optimized in our previous work. A total number of 14 singlet and 15 triplets were included at the ADC(2)/cc-pVDZ level, with the water molecules treated as fixed point charges. The range above 350 nm corresponds to the experimental window.

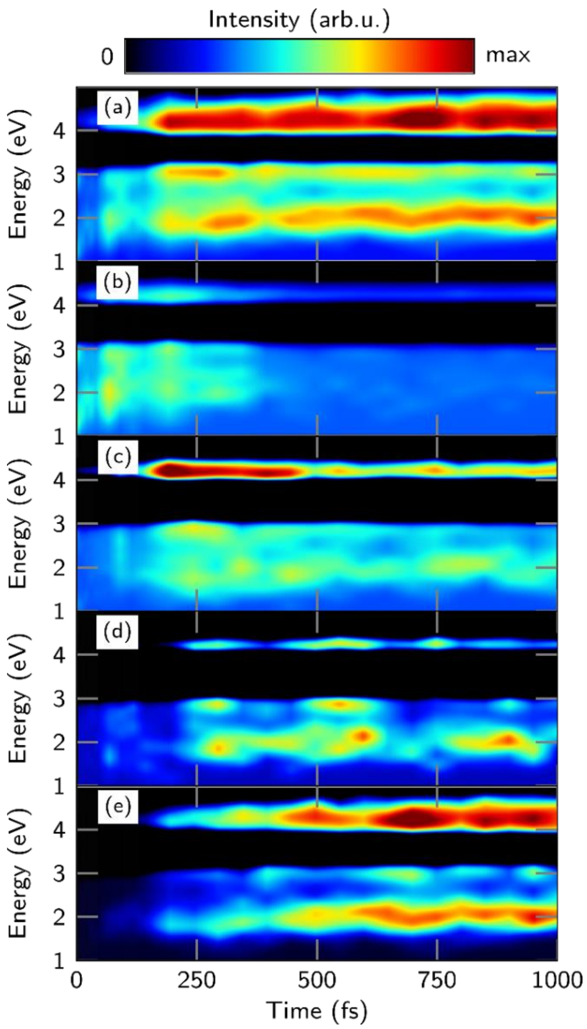

Figure S5: (a) Temporal evolution of TAS spectrum, convoluted with a 0.25 eV Gaussian. This spectrum was also decomposed in the adiabatic states (b) S_2, (c) S_1, (d) T_2 and (e) T_1.

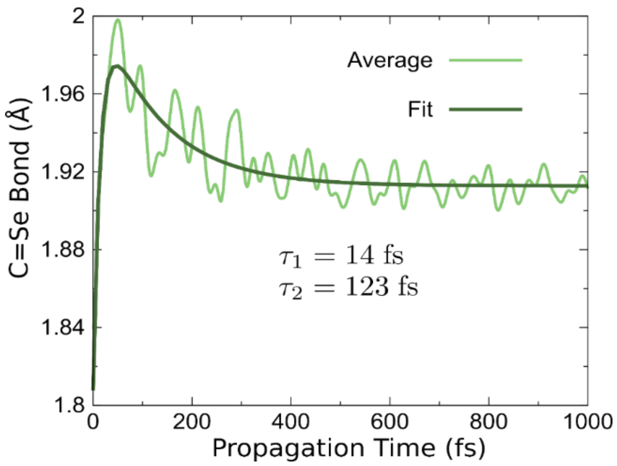

Figure S6: Temporal evolution of the C=Se bond length considering the average over an ensemble of 99 trajectories. Two time constants (τ_1 and τ_2) are extracted from a biexponential fit.

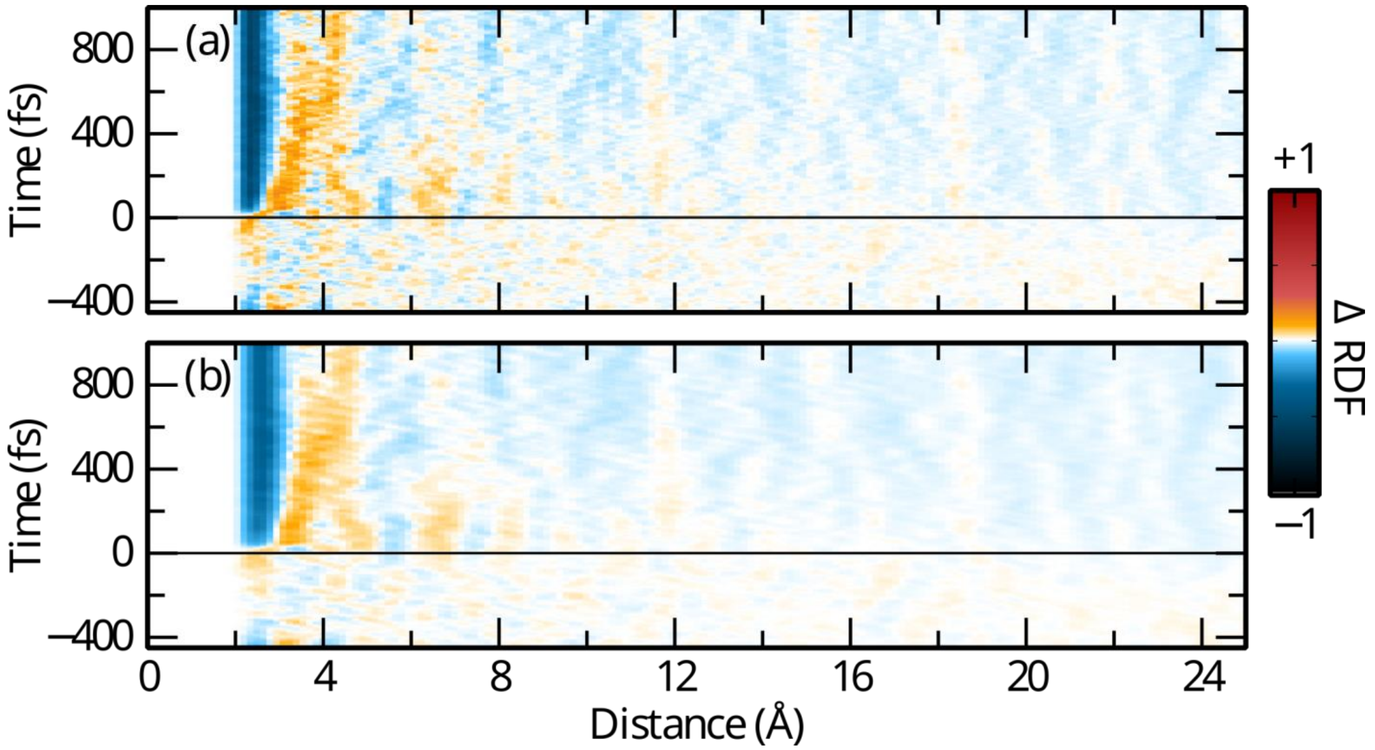

Figure S7: Difference between the radial distribution function (Se-H) in the excited and ground states with reduced spatial and temporal resolution. We used a bin width of (a) 0.15 Å, and (b) 0.15 Å × 10 fs.

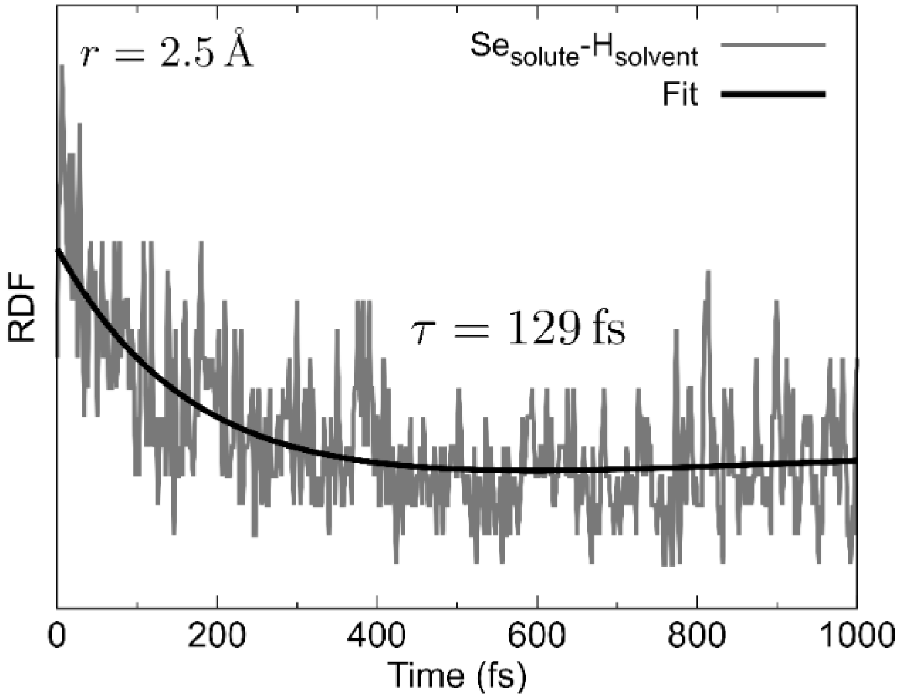

Figure S8: Temporal evolution of the radial distribution function between the selenium atom and the nearest hydrogen atoms of water. The maximum of the hydrogen bond shell is used as reference (r = 2.5 Å). The respective curve is fitted by a monoexponential fit.

Section S5. Optimized Geometries

```
16
 (S0)min MS-CASPT2
Se  -2.41392115  -0.09446381  -0.05771387
 N  +2.52823991  -1.54580031  -0.00843537
 C  +1.64215993  -2.60783470  +0.01584843
 N  +0.37420032  -2.23289960  +0.03436006
 C  +1.77376518  -0.40489425  -0.00366438
 N  +2.26026753  +0.87806291  -0.01378768
 C  +1.27476169  +1.75423171  -0.00963393
 N  +1.56265352  +3.10545464  -0.10315783
 N  -0.05889969  +1.42927955  +0.01609224
 C  -0.60775703  +0.12549388  +0.00594236
 C  +0.43878985  -0.84906403  +0.02486060
 H  +3.54197164  -1.58683789  -0.03160609
 H  +2.54998804  +3.27385995  +0.07879573
 H  +0.97155786  +3.70327220  +0.47121305
 H  -0.75085671  +2.17398965  -0.07854424
 H  +1.99135914  -3.64046995  +0.01889101
```

```
16
 (S1)min MS-CASPT2
Se  -2.38603941  -0.19497931  +0.44670914
 N  +2.51998510  -1.56186663  +0.03166725
 C  +1.63481111  -2.60540963  -0.01682706
 N  +0.36735516  -2.22177865  -0.09199502
 C  +1.77005287  -0.40225395  -0.01228040
 N  +2.24619454  +0.89679984  +0.06806579
 C  +1.26561119  +1.76416667  -0.00354167
 N  +1.54840598  +3.13090275  -0.06606686
 N  -0.08822890  +1.46705280  -0.07621189
 C  -0.59501227  +0.13778747  -0.19574665
 C  +0.44336312  -0.84010573  -0.09520040
 H  +3.53136799  -1.61186052  +0.09672183
 H  +2.53663075  +3.27356461  +0.13976048
 H  +0.97560815  +3.67474394  +0.57937230
 H  -0.66522826  +2.21729088  -0.45190006
 H  +1.97340411  -3.64267430  +0.00693326
```

```
16
 (S2)min MS-CASPT2
Se  -2.20222489  -0.01390148  +0.84750867
 N  +2.51281400  -1.55523237  +0.11167020
 C  +1.62835370  -2.60527310  -0.04972175
 N  +0.38299192  -2.23359095  -0.25655008
 C  +1.77388243  -0.40476842  +0.00495290
 N  +2.23333404  +0.86347632  +0.10279458
 C  +1.25761582  +1.75487324  -0.06458071
 N  +1.57561074  +3.10166734  -0.03055296
 N  -0.03589292  +1.42243826  -0.33577487
 C  -0.57923784  +0.12610874  -0.43451502
 C  +0.44200628  -0.84781228  -0.22773213
 H  +3.51029669  -1.60602556  +0.29599228
 H  +2.47729885  +3.24635613  +0.41956548
 H  +0.85492987  +3.70019289  +0.36835328
 H  -0.72446997  +2.17317373  -0.38913071
 H  +1.97097128  -3.64030350  -0.00281914
```

```
16
 (T2)min MS-CASPT2
Se  -2.34908699  -0.17464890  +0.51630268
 N  +2.51556250  -1.54970981  +0.04205936
 C  +1.63548410  -2.60941724  -0.01260641
 N  +0.36456356  -2.22174656  -0.10962013
 C  +1.76689204  -0.40167508  -0.01729673
 N  +2.24333143  +0.89013133   0.07312403
 C  +1.25713876  +1.76201100  -0.01557498
 N  +1.54475899  +3.12775713  -0.05826863
 N  -0.07971981  +1.45896914  -0.13145953
 C  -0.60030243  +0.13333628  -0.24585102
 C  +0.43766377  -0.84281678  -0.11852041
 H  +3.52514439  -1.59526925  +0.12760292
 H  +2.53223640  +3.26383301  +0.15483418
 H  +0.97616580  +3.66426726  +0.59776827
 H  -0.67065085  +2.21889055  -0.46309492
 H  +1.97909955  -3.64253186  +0.02006132
```

```
16
  (T1)min MS-CASPT2
Se  -0.3624732  +2.9749966  -0.9841418
 N  +2.2626147  -1.3313163  +0.2517938
 C  +3.1000200  -0.2477176  -0.0541628
 N  +2.5058486  +0.9107501  -0.1562829
 C  +0.9634585  -0.8470898  +0.3261468
 N  -0.1444215  -1.5117892  +0.4047716
 C  -1.3129638  -0.6517421  +0.4353357
 N  -2.5165683  -1.2138105  -0.0637892
 N  -1.2246846  +0.7401988  +0.1168399
 C  +0.0301656  +1.3474921  -0.0338321
 C  +1.1571920  +0.6090705  +0.1197290
 H  +2.5225466  -2.3097726  +0.2277789
 H  -2.5519449  -1.0737497  -1.0840954
 H  -2.4718117  -2.2250717  +0.0771783
 H  -1.9186801  +1.3154388  +0.5988673
 H  +4.1722320  -0.4027074  -0.1813211
```

```
16
  (S1/S2)CI MS-CASPT2
Se  -0.01954561   3.21113337  +0.16931489
 N  +2.29189226  -1.34057336  -0.16877520
 C  +3.13480670  -0.23230858  -0.10082173
 N  +2.51583098   0.91286217  +0.02557513
 C  +0.99480189  -0.88632170  -0.00331715
 N  -0.10406587  -1.58394257  +0.05131359
 C  -1.25207393  -0.81210858  +0.31003554
 N  -2.51361675  -1.28590917  -0.15879783
 N  -1.15292387  +0.61537100  +0.13196529
 C  +0.01509207  +1.30355432  +0.13378598
 C  +1.16966097  +0.54952418  +0.08982377
 H  +2.57234137  -2.31079344  -0.13291252
 H  -2.35493178  -1.92085134  -0.94499135
 H  -2.94754161  -1.85621941  +0.56607571
 H  -2.03551256  +1.10865654  +0.20586860
 H  +4.21494563  -0.34655815  -0.17268821
```

```
16
 (T1/T2)CI MS-CASPT2
Se  -0.02101422  +3.26462607  -0.51213701
 N  +2.27618645  -1.29901140  +0.14992472
 C  +3.12614808  -0.22736812  +0.06304680
 N  +2.50090760  +0.93096858  -0.03185909
 C  +1.00222006  -0.78347607  +0.10555566
 N  -0.18424504  -1.48960904  +0.14649639
 C  -1.22214226  -0.71403466  +0.03077270
 N  -2.53433278  -1.23101743  +0.11157311
 N  -1.20151267  +0.65573321  -0.16166286
 C  +0.00063336  +1.41228397  -0.02562225
 C  +1.16221883  +0.59453415  -0.01613565
 H  +2.52524884  -2.27080576  +0.22060767
 H  -2.46048025  -2.15792392  +0.51799490
 H  -2.91370647  -1.35279619  -0.82441380
 H  -2.04888251  +1.10222218  +0.16100928
 H  +4.20328168  -0.35114449  +0.06566855
```

```
16
 (S1/T2)ISC MS-CASPT2
Se  -2.37485910  -0.19720273  +0.48933663
 N  +2.52229543  -1.56043304  +0.02777800
 C  +1.63734589  -2.60563040  -0.01943709
 N  +0.36951964  -2.22291785  -0.09334178
 C  +1.76894856  -0.40211859  -0.01451386
 N  +2.24430203  +0.89924206  +0.06628136
 C  +1.26496231  +1.76326209  -0.00846044
 N  +1.54344559  +3.13155783  -0.06752711
 N  -0.08595144  +1.46482286  -0.08545088
 C  -0.59704210  +0.13604989  -0.19570607
 C  +0.44380579  -0.84038256  -0.09644092
 H  +3.53349626  -1.61297324  +0.08873441
 H  +2.53052415  +3.27907741  +0.14084362
 H  +0.96656237  +3.67346278  +0.57627524
 H  -0.66758426  +2.21635524  -0.45216518
 H  +1.97851010  -3.64079150  +0.00325411
```

```
16
 (S0/T1)ISC MS-CASPT2
Se  -1.76860934  -0.23699869  -1.14930039
 N  +2.44585469  -1.53658015  -0.15526963
 C  +1.58999810  -2.60670589  +0.09224804
 N  +0.34081374  -2.23082445  +0.32482235
 C  +1.70062887  -0.39822133  -0.07873265
 N  +2.17168924  +0.86022616  -0.18259275
 C  +1.21756651  +1.75929016  +0.10759086
 N  +1.59771823  +3.07953790  +0.18034328
 N  -0.05746990  +1.45643085  +0.42176463
 C  -0.67757017  +0.14611244  +0.42660395
 C  +0.37497201  -0.85073543  +0.21747859
 H  +3.44585108  -1.57580777  -0.33348753
 H  +2.51656369  +3.24825030  -0.21781040
 H  +0.89855752  +3.77518902  -0.06089659
 H  -0.66826702  +2.22685587  +0.68128281
 H  +1.94998890  -3.63463778  +0.08541553
```

```
16
 (S0)min ADC2
Se  -0.17918500  +3.19671282  +0.051905618
 N  +2.30223352  -1.28966496  -0.024076156
 C  +3.14071684  -0.19520950  +0.026067288
 N  +2.49300423  +0.96590151  +0.039791557
 C  +1.01963026  -0.80243374  -0.043279495
 N  -0.12135577  -1.54732593  -0.108700087
 C  -1.19296117  -0.77906491  -0.100784990
 N  -2.45659857  -1.35590656  -0.074108696
 N  -1.15909069  +0.59303643  -0.044854252
 C  -0.01788506  +1.41213246  -0.004679406
 C  +1.16348976  +0.59844102  -0.004180188
 H  +2.56495606  -2.27047355  -0.041411876
 H  -2.39412105  -2.34487367  -0.310146747
 H  -3.14222348  -0.88431139  -0.661878773
 H  -2.04057891  +1.10521211  +0.014571500
 H  +4.22495513  -0.30746643  +0.051315954
```

```
16
 (S1)min ADC2
Se  -2.39905509  -0.20000224  +0.35113155
 N  +2.52178071  -1.55911347  +0.02902744
 C  +1.64202787  -2.61062701  -0.00556379
 N  +0.36086982  -2.22209399  -0.07291166
 C  +1.76870884  -0.40349569  -0.02016039
 N  +2.24244453  +0.89689986  +0.04289205
 C  +1.26323746  +1.76545729  -0.00722377
 N  +1.54656565  +3.13397601  -0.07515929
 N  -0.09155125  +1.47276842  -0.03405548
 C  -0.58980276  +0.13918727  -0.17438292
 C  +0.44195116  -0.84660324  -0.08405512
 H  +3.53435781  -1.60821100  +0.08256607
 H  +2.54057630  +3.27246373  +0.10620515
 H  +0.99644173  +3.67399270  +0.59378672
 H  -0.68014004  +2.22354178  -0.39388532
 H  +1.97986848  -3.64676018  +0.02124880
```

```
16
 (S2)min ADC2
Se  -2.31294026  -0.07278211  +0.582068980
 N  +2.52317830  -1.55332564  +0.078469477
 C  +1.64097018  -2.61008643  -0.010018355
 N  +0.37235858  -2.23416455  -0.168506569
 C  +1.77584178  -0.40542028  -0.024095036
 N  +2.24182618  +0.87304593  +0.044312477
 C  +1.26368176  +1.76222998  -0.057556718
 N  +1.58055735  +3.11620332  -0.049446297
 N  -0.05452617  +1.43918859  -0.220739760
 C  -0.57751687  +0.12885390  -0.385516331
 C  +0.43824932  -0.85618888  -0.180250600
 H  +3.52832747  -1.59907677  +0.216693903
 H  +2.51701270  +3.25143590  +0.328469906
 H  +0.90280706  +3.69635473  +0.443160605
 H  -0.74342546  +2.18946206  -0.287394128
 H  +1.98187810  -3.64435075  +0.049808464
```

```
16
 (T2)min ADC2
Se  -0.09593989  +3.27984418  -0.00274987
 N  +2.33418862  -1.27972002  +4.00000000
 C  +3.16894380  -0.19176993  +0.00059160
 N  +2.50895394  +0.97648989  +0.00007466
 C  +1.03914055  -0.79807236  +0.00021984
 N  -0.12258279  -1.55268024  +0.00017705
 C  -1.19284686  -0.78881218  +0.00007671
 N  -2.42866914  -1.38491451  +0.00033547
 N  -1.19891668  +0.59349024  -0.00015372
 C  -0.00513277  +1.37646732  -0.00045620
 C  +1.18364064  +0.59587374  -0.00016615
 H  +2.60298240  +2.25831967  +0.00126695
 H  -2.45102088  -2.39503147  +0.00011491
 H  -3.29119430  -0.86311367  -0.00075477
 H  -2.09505380  +1.06981490  +0.00064289
 H  +4.25403421  -0.29636363  +0.00081905
```

```
16
 (T1)min ADC2
Se  -2.08447019  -0.27353271  +1.40364272
 N  +2.51066730  -1.55219629  -0.07659055
 C  +1.64401229  -2.60806270  +0.07077911
 N  +0.37232408  -2.22226273  +0.22614837
 C  +1.75738463  -0.40132649  -0.00658581
 N  +2.23316496  +0.89281763  -0.07425052
 C  +1.24943053  +1.76128687  +0.01786499
 N  +1.51748475  +3.12347366  -0.14798148
 N  -0.07810978  +1.45905254  +0.22259407
 C  -0.61922814  +0.13204050  +0.24554755
 C  +0.44060161  -0.84650903  +0.17243650
 H  +3.51583938  -1.59599499  -0.21272976
 H  +2.52748311  +3.26317781  -0.13539332
 H  +1.08057410  +3.69774125  +0.57346029
 H  -0.73197015  +2.22276533  +0.06094963
 H  +1.98764751  -3.64242741  +0.05542068
```

```
16
 (S1/S2)CI ADC2
Se  -0.08236021  +3.18377822  -0.01402447
 N  +2.29095491  -1.35589810  -0.11824258
 C  +3.12973365  -0.24263809  -0.10857115
 N  +2.52508830   0.91802719  +0.03251710
 C  +0.99221728  -0.90715806  +0.07622155
 N  -0.09471575  -1.60117240  +0.11949325
 C  -1.27314710  -0.80513742  +0.38251289
 N  -2.53311244  -1.27174806  -0.10355812
 N  -1.15265747  +0.61324481  +0.21505107
 C  +0.02094197  +1.30475230  +0.14634417
 C  +1.17809764  +0.56770415  +0.14708625
 H  +2.56678702  -2.32944503  -0.19867614
 H  -2.82883847  -2.06054517  +0.47711543
 H  -2.39690249  -1.67278611  -1.04295877
 H  -2.02354764  +1.14681028  +0.20221630
 H  +4.21062072  -0.36227321  -0.21107230
```

```
16
   (T1/T2)CI ADC2
Se  -0.09277529  +3.03712519  -0.97004161
 N  +2.29743659  -1.28898149  +0.14834821
 C  +3.14741005  -0.21146509  +0.09724211
 N  +2.50675457  +0.96259778  +0.04821441
 C  +1.01567997  -0.78585369  +0.12657437
 N  -0.14450240  -1.52303978  +0.11323399
 C  -1.20883943  -0.74060527  +0.08983151
 N  -2.47884030  -1.31758049  +0.16730733
 N  -1.18229174  +0.62703833  +0.05575020
 C  -0.00293066  +1.44367605  +0.08302250
 C  +1.17652625  +0.61225992  +0.06865964
 H  +2.54951384  -2.27179767  +0.18252132
 H  -2.39565571  -2.32193023  +0.01304501
 H  -3.13723717  -0.91659479  -0.50070978
 H  -2.07058218  +1.11054004  +0.17727618
 H  +4.23086231  -0.33220773  +0.10054373
```

```
16
(S1/T2)ISC ADC2
Se  -0.03546535  +3.17677719  -0.74613974
 N  +2.30374421  -1.28645702  +0.17385889
 C  +3.14806281  -0.20936692  +0.09027103
 N  +2.50038034  +0.96007561  -0.01432136
 C  +1.01533525  -0.79185253  +0.12263729
 N  -0.15981032  -1.52895018  +0.12634352
 C  -1.21121132  -0.75514099  +0.05273274
 N  -2.49388820  -1.30099413  +0.17169790
 N  -1.21285836  +0.62688184  -0.11052579
 C  -0.00750923  +1.39446046  -0.04703976
 C  +1.17166609  +0.59330743  +0.00823603
 H  +2.56404059  -2.26410905  +0.25746143
 H  -2.42399247  -2.31772137  +0.13370752
 H  -3.12085640  -0.96977709  -0.56240879
 H  -2.05920875  +1.07953618  +0.23640489
 H  +4.23209981  -0.32348836  +0.10790333
```

```
16
(S0/T1)ISC
Se  -1.47442428  -0.26403480  +1.84471081
 N  +2.47066836  -1.54582927  +0.02103597
 C  +1.58555377  -2.60697132  -0.02705386
 N  +0.31196983  -2.23124053  +0.02201422
 C  +1.71956805  -0.40318919  +0.10609467
 N  +2.20485355  +0.87307137  +0.11484789
 C  +1.21812362  +1.75856477  +0.01821891
 N  +1.55054748  +3.09974938  -0.12832782
 N  -0.10948530  +1.45525749  -0.03398721
 C  -0.70461123  +0.14706467  +0.13773775
 C  +0.37349667  -0.84946771  +0.11167416
 H  +3.48456349  -1.58353007  -0.02332630
 H  +2.53880178  +3.23522676  +0.07819292
 H  +0.97150498  +3.73110144  +0.42312218
 H  -0.75134826  +2.23233343  -0.17347175
 H  +1.93305348  -3.63806318  -0.09617006
```

References

[S1] S. Mai, A. Wolf, L. González. Curious Case of 2-Selenouracil: Efficient Population of Triplet States and Yet Photostable. *J. Chem. Theory Comput.* **2019**, 15 (6), 3730-3742.

[S2] K. M. Farrell, M. M. Brister, M. Pittelkow, T. I. Sølling, C. E. Crespo-Hernández. Heavy-Atom-Substituted Nucleobases in Photodynamic Applications: Substitution of Sulfur with Selenium in 6-Thioguanine Induces a Remarkable Increase in the Rate of Triplet Decay in 6-Selenoguanine. *J. Am. Chem. Soc.* **2018**, 140 (36), 11214-11218.

[S3] D. Cremer, J. A. Pople. General definition of ring puckering coordinates. *J. Am. Chem. Soc.* **1975**, 97 (6), 1354-1358.

[S4] J. C. A. Boyens. The conformation of six-membered rings. *J. Cryst. Mol. Struct.* **1978**, 8, 317–320.

[S5] Y. Fang, Q. Peng, Q. Fang, W. Fang, G. Cui. MS-CASPT2 Studies on the Photophysics of Selenium-Substituted Guanine Nucleobase. *ACS Omega* **2019**, 4 (6), 9769-9777.

[S6] S. Battaglia, R. Lindh. Extended Dynamically Weighted CASPT2: The Best of Two Worlds.

J. Chem. Theory Comput. **2020**, 16 (3), 1555-1567.

[S7] Y. Fang, D. Valverde, S. Mai, S. Canuto, A. C. Borin, G. Cui, L. González. *J. Phys. Chem. B* **2021**, 125 (7), 1778-1789.
